# Investigation of brain connectivity alteration in the non-severe traumatic brain injury: an emotional condition study

**DOI:** 10.7717/peerj.21593

**Published:** 2026-08-05

**Authors:** Hazim Omar, Aini Ismafairus Abd Hamid, Muhammad Abdul Rahman, Nor Azila Noh, Hamwira Sakti Yaacob, Wen Jia Chai, Zamzuri Idris, Asma Hayati Ahmad, Diana Noma Fitzrol, Ab. Rahman Izaini Ab. Ghani, Nurfaten Hamzah, Wan Nor Azlen Wan Mohamad, Mohamed Faiz Mohamed Mustafar, Muhammad Hafiz Hanafi, Mohammed Faruque Reza, Hafidah Umar, Mohd Faizal Mohd Zulkifly, Song yee Ang, Zaitun Zakaria, Kamarul Imran Musa, Azizah Othman, Zunaina Embong, Nur Asma Sapiai, Regunath Kandasamy, Haidi Ibrahim, Mohd Zaid Abdullah, Pedro Antono Valdes-Sosa, Maria Luisa Bringas Vega, Bharat Biswal, Putra Sumari, Paramjit Singh Jamir Singh, Jafri M. Abdullah

**Affiliations:** 1 Department of Neurosciences & Brain Behaviour Cluster, Hospital Pakar Universiti Sains Malaysia, Universiti Sains Malaysia, Kota Bharu, Kelantan, Malaysia; 2 Brain Behaviour Cluster and Department of Neurosciences, School of Medical Sciences, Universiti Sains Malaysia, Kota Bharu, Kelantan, Malaysia; 3 School of Medical Imaging, Faculty of Health Sciences, Universiti Sultan Zainal Abidin, Kuala Nerus, Terengganu, Malaysia; 4 Faculty of Medicine and Health Sciences, Universiti Sains Islam Malaysia, Nilai, Negeri Sembilan, Malaysia; 5 Department of Computer Science, Kulliyah of Information and Communication Technology, International Islamic University, Kuala Lumpur, Malaysia; 6 Department of Psychology, Faculty of Social Sciences, Raffles University, Iskandar Puteri, Johor, Malaysia; 7 Department of Physiology, School of Medical Sciences, Universiti Sains Malaysia, Kota Bharu, Kelantan, Malaysia; 8 Dr. Sid E. Williams Center for Chiropractic Research, Life University, Georgia, United States of America; 9 Department of Community Medicine, School of Medical Sciences, Universiti Sains Malaysia, Kota Bharu, Kelantan, Malaysia; 10 Department of Paediatrics, School of Medical Sciences, Universiti Sains Malaysia, Kota Bharu, Kelantan, Malaysia; 11 Department of Ophthalmology, School of Medical Sciences, Universiti Sains Malaysia, Kota Bharu, Kelantan, Malaysia; 12 Department of Radiology, School of Medical Sciences, Universiti Sains Malaysia, Kota Bharu, Kelantan, Malaysia; 13 Neuroscience Clinic, Gleneagles Hospital, Kuala Lumpur, Malaysia; 14 School of Electrical and Electronic Engineering, Universiti Sains Malaysia, Nibong Tebal, Malaysia; 15 The Clinical Hospital of Chengdu Brain Science Institute, MOE Key Lab for Neuroinformation, University of Electronic Science and Technology of China, Chengdu, China; 16 The Cuban Neurosciences Center, Havana, Cuba; 17 Department of Biomedical Engineering, New Jersey Institute of Technology, Newark, NJ, United States of America; 18 School of Computer Sciences, Universiti Sains Malaysia, Gelugor, Penang, Malaysia; 19 School of Social Sciences, Universiti Sains Malaysia, Gelugor, Penang, Malaysia

**Keywords:** Traumatic brain injury, Functional connectivity, Effective connectivity, Emotion, Neuropsychology, Emotion network, IAPS, Brain activation

## Abstract

**Background:**

Traumatic brain injury (TBI) frequently results in persistent emotional and cognitive dysfunction, yet the neural mechanisms underlying these deficits remain incompletely understood. This study examined the impact of non-severe TBI (nTBI) on emotional processing, with a focus on brain activation, functional connectivity, effective connectivity, and their relationship to cognitive performance.

**Methods:**

Twenty right-handed male nTBI participants (mean age: 30.70 ± 11.78 years) and 20 age-matched healthy male controls (mean age: 27.85 ± 7.73 years) underwent functional magnetic resonance imaging (fMRI) while viewing emotional images from the International Affective Picture System (IAPS). Brain activation was analyzed using Statistical Parametric Mapping (SPM12). Functional connectivity was assessed with seed-based analysis (medial visual network, mVN) using the CONN toolbox. Effective connectivity was evaluated using dynamic causal modeling (DCM) and parametric empirical Bayes (PEB). Neuropsychological assessments covering memory, intelligence, and executive function were included to characterize general cognitive function within the cohort, with exploratory analyses examining relationships with connectivity measures.

**Results:**

Compared to controls, the nTBI group exhibited reduced activation in the calcarine cortex and lingual gyrus during emotional processing. Functional connectivity analysis showed decreased mVN connectivity with the frontal pole and dorsal anterior cingulate cortex (dACC), with increased mVN-ACC and mVN-supramarginal gyrus connectivity specifically during negative emotion processing. Effective connectivity analysis revealed altered directional influences within visual-limbic-cognitive control networks, including altered mVN-to-dACC and right amygdala-to-dACC connectivity patterns, as well as valence-specific modulation of self-inhibition. Exploratory analyses suggested potential correlations between connectivity alterations and cognitive performance, though these relationships varied across domains and should be interpreted cautiously.

**Conclusions:**

Non-severe TBI disrupts emotional network function by altering both functional and effective connectivity, particularly in visual, limbic, and cognitive control regions. These alterations may contribute to emotional dysregulation following injury. Findings support the relevance of brain connectivity-based markers in understanding post-TBI emotional processing and highlight potential targets for intervention. Interpretations should be made cautiously, given the study limitations, including the use of static visual stimuli and a homogenous male sample.

## Introduction

Traumatic brain injury (TBI), including non-severe TBI (nTBI), affects millions of people worldwide and often leaves a hidden legacy of emotional and cognitive challenges. While the immediate physical consequences of nTBI may be less apparent compared to more severe injuries, the disruption to the complex brain emotional circuitry can significantly alter an individual’s life. This impact can affect their relationships, overall well-being and ability to navigate daily life. A common and induced effect of TBI is emotional dysregulation, which may demonstrate increased emotional responses, mood swings, and impaired emotional regulation. One crucial aspect of this dysregulation is a significant impairment in the ability to recognize and interpret the emotions of others. Research involving TBI has shown that individuals with TBI often experience substantial difficulties in emotion recognition, particularly related to negative emotions such as fear and anger. A systematic review by Murphy et al. (2022) of 17 studies found that the TBI group demonstrated moderate to large deficits (Hedges’ g = 0.79) in recognizing emotions compared to the healthy group, with the most pronounced impairments occurring for negative emotions. These impairments extend across various emotional categories and modalities, including across facial and vocal cues (Murphy et al., 2022).

Moreover, TBI frequently affects cognitive functions essential for emotional well-being, including memory, executive functions, and self-awareness (Cerino et al., 2025; Gerber et al., 2025; Villalobos, Bivona & Botella, 2025). These cognitive deficits can directly lead to emotional challenges by impairing an individual’s capacity to recognize and manage emotions and engage effectively in social interactions. In addition to these direct effects, the cognitive and emotional consequences of nTBI can contribute to long-term psychological challenges. Conditions such as post-traumatic depression (PTD), characterized by persistent sadness, anxiety, and impaired concentration, are prevalent (Bombardier et al., 2016) and significantly reduce daily function and quality of life. Furthermore, 60% of participants with nTBI report reduced empathy (Hillis, 2014), further complicating social relationships and intensifying feelings of isolation.

Advancements in neuroimaging technologies, particularly functional magnetic resonance imaging (fMRI) and dynamic causal modeling (DCM), have significantly enhanced our understanding of the neural mechanisms that underlie cognitive and emotional deficits following moderate TBI, revealing altered patterns of brain activity and connectivity (McDonald et al., 2021; Mohamed et al., 2022). These techniques enable researchers to visualize brain activity and the connectivity patterns involved in emotional processing, revealing notable disruptions in critical brain regions and networks (Gimbel et al., 2023). fMRI studies have consistently shown altered neural activity in the amygdala, a key area responsible for processing emotions such as fear and pleasure, as well as in the prefrontal cortex, which plays a vital role in executive functions and emotional regulation. Additionally, DCM research has emphasized the significance of effective connectivity and how one brain region influences another when examining the effects of TBI on cognitive and emotional processing. Findings indicate changes in connectivity between the prefrontal cortex and limbic structures, which likely contribute to the emotional dysregulation commonly experienced by individuals suffering from moderate TBI (Sours et al., 2015).

This study investigates the effects of nTBI on emotional processing using fMRI, with analyses including Statistical Parametric Mapping 12 (SPM12) for brain activation, the CONN toolbox for seed-based functional connectivity, and DCM with PEB for effective connectivity. We aim to identify alterations in neural activity and directional connectivity within and between brain networks involved in visual processing, emotion, and cognitive control that contribute to emotional and cognitive deficits following nTBI. We hypothesize that, compared to healthy controls (HC), participants with nTBI will exhibit reduced activation in visual processing regions during emotional processing, altered effective connectivity within a network encompassing the medial visual network (mVN), amygdala, and dorsal anterior cingulate cortex (dACC), and significant correlations between these neural alterations and impairments in neuropsychological performance, specifically in domains of general intelligence, memory, and executive function. In addition, exploratory analyses were conducted to examine potential relationships between neural connectivity alterations and neuropsychological performance across domains of general intelligence, memory, and executive function. These analyses were intended to provide contextual brain–behaviour information and were not part of the primary hypothesis-driven objective.

This article is structured as follows: ‘Materials & Methods’ details the materials and methods, including participant recruitment, fMRI paradigm using affective stimuli, data acquisition procedures, and analysis techniques (SPM12, CONN, and DCM-PEB). ‘Results’ reports the results of brain activation, functional and effective connectivity, along with associated neuropsychological outcomes. ‘Discussion’ discusses the findings in the context of emotional network alterations post-nTBI. ‘Conclusions and limitations’ concludes with a summary, study limitations, and directions for future research.

## Materials & Methods

### Participant

The study enrolled twenty healthy male controls (mean age ± SD = 27.85 ± 7.73) and 20 male participants with nTBI (nTBI; mean age ± SD = 30.70 ± 11.78; Glasgow Coma Scale (GCS): median = 14.5 (median was used as the data is ordinal in nature)) were recruited. The nTBI participants were recruited from the Department of Emergency, Hospital Pakar Universiti Sains Malaysia, during the subacute phase (4–6 weeks post-injury) and met the criteria for nTBI (GSC score of 8 to 15). Exclusion criteria included prior nTBI, psychiatric or neurological disorders, substance misuse, or MRI contraindications. All participants were right-handed, as confirmed by the Edinburgh Inventory (Oldfield, 1971) and reported no current use of psychoactive medications. Male-only recruitment was used to reduce biological variability related to known sex differences in brain structure, hormonal influences, and functional connectivity patterns (citations), particularly given the modest sample size of this network-level neuroimaging study. Written informed consent was obtained from all participants, and the study protocol was approved by the Human Research Ethics Committee Universiti Sains Malaysia (USM/JEPeM/15110485 and USM/JEPeM/20080406). The research was conducted in accordance with the Declaration of Helsinki.

### Neuropsychological assessment

A subset of participants (11 HC and 19 nTBI) underwent neuropsychological assessments to evaluate cognitive domains commonly impaired after nTBI. General cognitive ability was assessed using the Wechsler Abbreviated Scale of Intelligence, 1st edition (WASI; block design and matrix reasoning) (McCrimmon & Smith, 2013), verbal memory with the Rey Auditory Verbal Learning Test (RAVLT; immediate and delayed recall) (Bean, 2018; Khosravi Fard et al., 2016) and visual memory and perception with the Rey Complex Figure Test and Recognition Trial (RCFT; immediate and delayed recall) (Sargénius et al., 2017), respectively. Psychomotor speed and cognitive flexibility were measured *via* the Comprehensive Trail-Making Test (CTMT) (Beratis et al., 2018; Gray, 2006), while executive functioning was evaluated using the Wisconsin Card Sorting Test (WCST) (Kopp, Lange & Steinke, 2021). Scores were standardized for age and education. For all the test used in this study, the authors have permission to use this instrument from the copyright holders. Although the study initially recruited 20 participants per group, only a subset (11 HC and 19 nTBI) completed the neuropsychological assessments. This reduction was primarily due to logistical constraints such as the COVID-19 movement restriction order, inability to contact some participants, and voluntary withdrawal particularly among healthy controls. Additionally, neuropsychological testing was scheduled separately from the fMRI session, typically with a one- to two-day gap to allow sufficient rest and avoid fatigue-related confounds. The neuropsychological battery focused on global cognitive domains rather than affective symptom measures, as the primary objective of this study was to characterise neural network alterations associated with emotional stimulus processing rather than clinical affective symptom severity. These measures were included to characterise general cognitive function in the cohort. Correlation analyses with neuroimaging measures were exploratory and were not part of the primary study objective.

### Stimulus and task design

**Figure 1:**
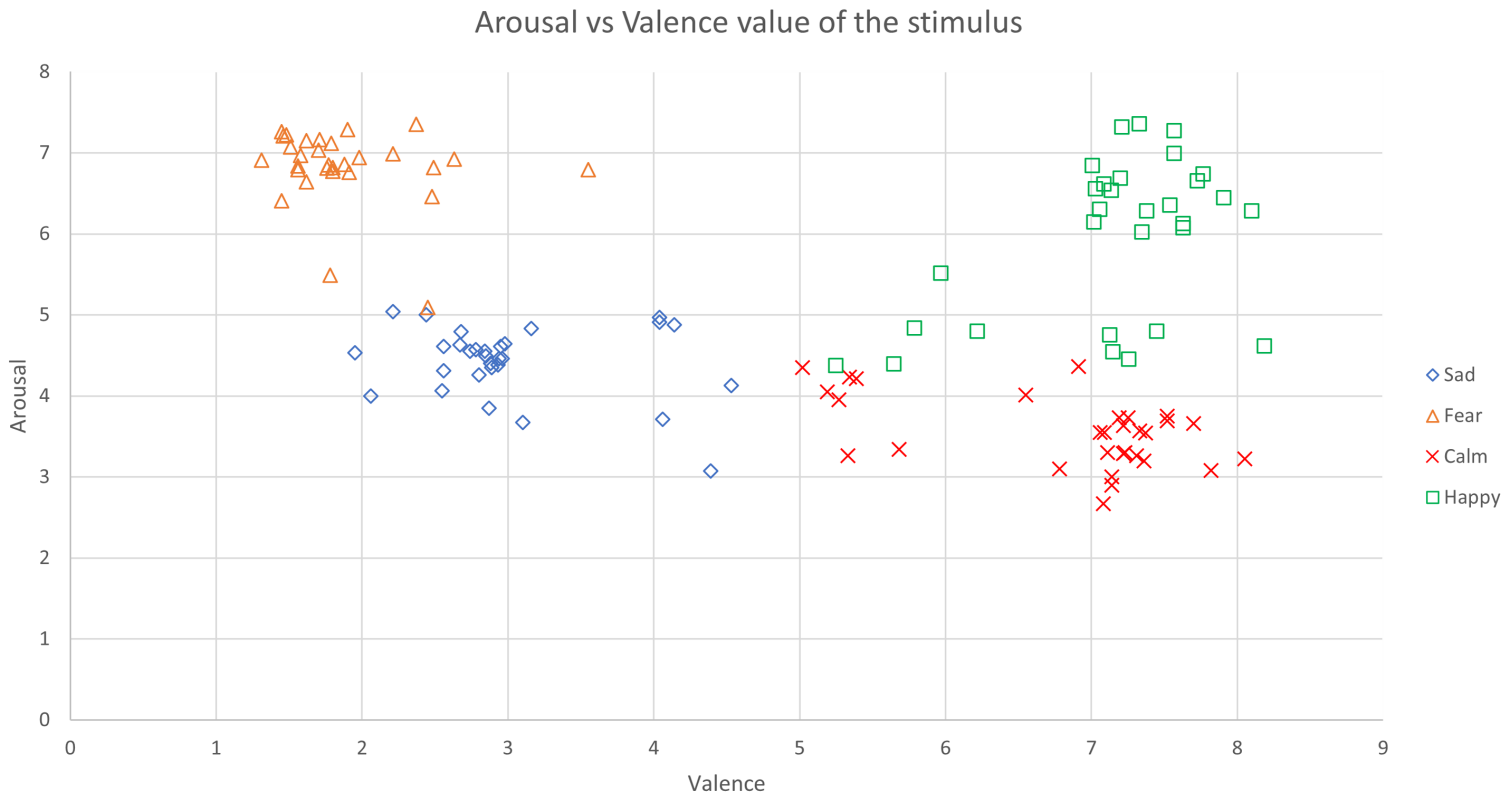
Scatterplot of the arousal and valence ratings for the IAPS pictures used in this study categorized by emotional condition.

The emotional paradigm employed a block design implemented using E-prime 1.1 software, configured at a resolution of 1,280  ×  1,024 with a 32-bit color depth. The stimuli consisted of 120 images selected from the International Affective Picture System (IAPS) (Lang, Bradley & Cuthbert, 2008) and were categorized into four emotional conditions based on normative valence and arousal ratings: sad (negative valence, low arousal), fear (negative valence, high arousal), calm (positive valence, low arousal), and happy (positive valence, high arousal). The average valence and arousal values for each condition are presented in Table 1 and the illustrated scatterplot is presented in Fig. 1.

**Figure 2:**
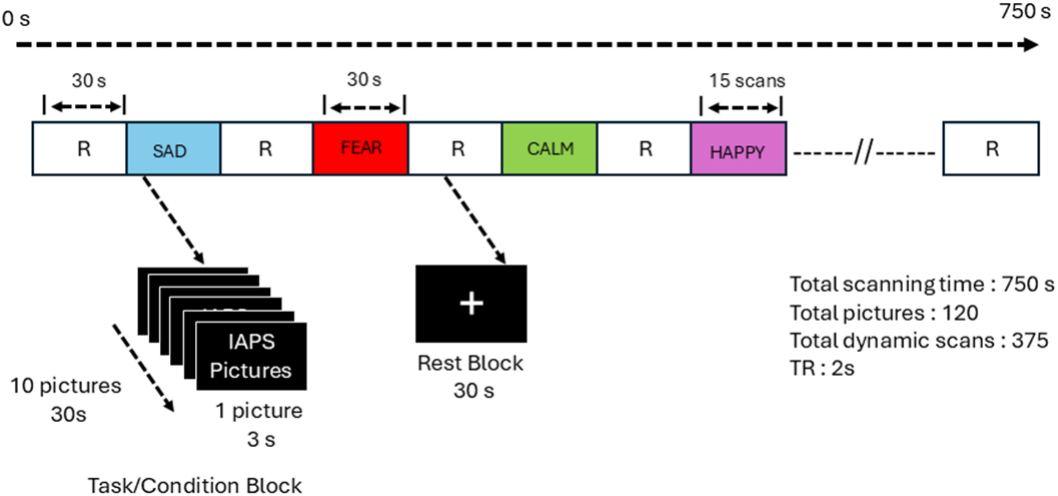
Block design emotional task paradigm used in the fMRI experiment. Image stimuli were from the IAPS (Lang, Bradley & Cuthbert, 2008). Abbreviation: R, Resting block; TR, Time repetition; s, seconds.

Each condition comprised 30 images divided into three blocks. Within each block, images from the same emotional condition were presented consecutively for 3 s each, resulting in 10 images per block (30 s per block). Blocks were interspersed with 30-second rest intervals, during which a white fixation cross was displayed against a black background. The task was projected onto an MRI-compatible screen and viewed through a back-reflection mirror, with a total stimulus presentation time of 750 s (12.6 min). A simplified illustration of the block design is depicted in Fig. 2. Stimuli were presented in a pseudo-randomised order to minimize predictability.

**Table 1:** The average valence and arousal ratings for each experimental condition.

**Condition**	**Average valence (SD)**	**Average arousal (SD)**
**Sad**	3.018 (0.666)	4.424 (0.442)
**Fear**	1.885 (0.473)	6.826 (0.478)
**Calm**	6.806 (0.887)	3.549 (0.436)
**Happy**	7.144 (0.707)	5.951 (0.975)

### fMRI data acquisition and preprocessing

MRI data were acquired with a Philips Achieva 3.0 T MRI scanner equipped with a 32-channel head coil. For each participant, functional runs T2*-weighted echo-planar imaging (EPI) with a 64  ×  62 matrix, 35 axial slices were obtained parallel to the anterior commissure-posterior commissure (AC-PC) line. The imaging parameters included a repetition time (TR) of 2,000 ms, echo time (TE) of 30 ms, and flip angle (FA) of 78°. The field of view for functional images was 192 mm × 192 mm × 105 mm, with a slice thickness of three mm with no inter-slice gap. The slice acquisition was interleaved, resulting in a total of 375 scans acquired over 12.6 min, yielding a voxel size of 1.7  × 1.7  × 3 mm. Additionally, a high-resolution T1-weighted anatomical image was acquired using the MPRAGE sequence, characterized by a 240  × 240 matrix and 240 sagittal slice volumes. The parameters for the anatomical imaging included a TR of 7.5 ms, TE of 3.46 ms, FA of 8° and a voxel size of 1  × 1  × 1.2 mm. These setups ensure the optimal sensitivity to BOLD contrast, facilitating the detection of neural activity associated with emotional processing (Ferraro et al., 2025; Landsiedel & Koldewyn, 2023). During the scanning session, participants were instructed to remain still while observing the images displayed on a screen through a reflective mirror mounted on the head coil.

To mitigate potential MRI signal instability, the initial 10 volumes of the fMRI data were discarded before preprocessing (Li et al., 2019). Data preprocessing was conducted utilizing the Statistical Parametric Mapping 12 (SPM12) software package, implemented in MATLAB (*v.* R2021a), following a standard pipeline. Functional data underwent slice timing correction and realignment to correct the motion artifacts. Following this, the participant’s T1-weighted anatomical image was coregistered to the mean functional images to maximize mutual information between the structural and functional data, ensuring accurate alignment. The anatomical images were then segmented and normalized to standard space using the SPM12 Montreal Neurological Institute (MNI) template. The resulting normalization parameters were applied to the functional data. Finally, the normalized functional data were spatially smoothed using a Gaussian kernel with a full width at half maximum (FWHM) of eight mm. Outlier scans were detected using the Artifact Detection Toolbox (ART) three implemented in MATLAB (The MathWorks, Natick, MA, USA). The threshold for global mean detection was set at three standard deviations (SD) with a movement threshold of 0.5 mm (Dailey et al., 2018). This approach corresponds to a conservative 95% confidence interval, ensuring robust detection of artifacts. To support replicability, the raw data for this study is available as Supplementary Material (Omar et al., 2025).

### Statistical analysis of fMRI brain activations

Statistical analyses were conducted using Statistical Product and Service Solutions (SPSS 29; IBM Corp., Armonk, NY, USA) to evaluate demographic data, neuropsychological evaluations, and correlational analyses. Demographic and neuropsychological data were analyzed between groups employing an independent *t*-test with a significance threshold set at 0.05. Levene’s test was utilized to assess the equality of variance. Following these statistical analyses, effect size calculations were performed to evaluate the magnitude of differences or relationships observed across groups. Corrected Cohen’s d (Hedge’s g) was used to estimate the effect size, which aids in result interpretation given the variance in group sizes (Gerchen, Kirsch & Feld, 2021; Pernet et al., 2021). An effect size of 0.2 is considered to be a minor effect, 0.5 is considered to be a medium effect, and 0.8 is considered to be a high effect (Lakens, 2013).

First-level analyses for each participant were executed using multiple linear regression, incorporating four explanatory variables that corresponded to the four stimulus classes of interest: sad, fear, calm, and happy conditions. Each condition block was modelled by convolving a boxcar function, representing the duration of the block with a canonical hemodynamic response function (HRF), thereby generating regressors of interest for the linear model. Additionally, six parameters derived from the motion correction algorithm were included as confounding regressors. Within this first-level analysis, linear contrasts were employed to compare condition-specific effects (*e.g.*, Fear, Happy, *etc.*) and to produce contrast images for subsequent second-level analysis.

For the second level analysis, a full factorial design comprised two groups (Healthy and nTBI) and the four aforementioned emotional conditions. The same general linear model (GLM) approach was applied to identify significant brain activation patterns across various comparisons. A stringent family wise error (FWE) activation threshold was used at the peak voxel and cluster activation threshold level (p_*fwe*_ < 0.05) for generating group-level statistical maps (Hartling et al., 2021).

### Functional connectivity analysis

The functional connectivity analysis was conducted using the CONN Toolbox (v.20b; RRID: SCR_009550), an open-source software package based on MATLAB that is compatible with SPM12 data formats (Whitfield-Gabrieli & Nieto-Castanon, 2012). The images were preprocessed and denoised using component-based noise reduction (CompCor). This method eliminated signals from white matter and cerebrospinal fluid and addressed outlier scans associated with motion artifacts. The functional outlier detection was established with a conservative threshold at the 95th percentile of the normative sample, with a target structural resolution of one mm and a functional resolution target of two mm. The smoothing kernel was applied at six mm FWHM.

Subsequently, both seed-based and region-of-interest (ROI)-based functional connectivity (FC) analyses were performed. In the seed-based analysis, seed regions were selected based on the brain activation analysis results derived from the main effect of group. For the ROI-based FC analysis, all predefined ROIs within the CONN toolbox were utilized. Multiple comparisons were corrected using a threshold of false discovery rate (FDR)-corrected *p*-value being less than 0.05 (p_*FDR*_ < 0.05) at the cluster level and p_*uncorrected*_ < 0.001 at the voxel level (Sours et al., 2017).

### Dynamic causal modelling method

DCM was performed using SPM12, which is a Bayesian framework designed to infer directed (effective) connectivity among brain regions (Kiebel, Garrido & Friston, 2007; Yang et al., 2020). The primary aim of the present study was to investigate the alterations in neural circuitry as well as affective and emotional responses between healthy participants and those with TBI. To achieve this, the DCM model was developed based on the prior findings from brain activation and functional connectivity analyses.

In the preceding section on brain activation, significant differences were observed only for the fear and happy conditions, with all significant conditions categorizing with the same arousal quadrant (high arousal; see Table S1). Consequently, the conditions were reclassified for the DCM analysis according to their valence. Specifically, sad and fear conditions were combined to represent a negative valence, whereas calm and happy were categorized as a positive valence. This recategorization aligns with the framework established in earlier research focusing on the dimensional aspects of valence (Anders et al., 2004; Feng et al., 2012; Pirastru et al., 2023). This methodological approach not only offers computational efficiency but also improves statistical accuracy.

Group-level inference on effective connectivity parameters was conducted using PEB, a hierarchical Bayesian approach for analysing DCM parameters at the group level (Friston et al., 2016; Zeidman et al., 2019a; Zeidman et al., 2019b). This method carries both the estimated connection strengths and their associated uncertainties from individual participants into the group analysis, so that less reliable data contribute less to the final group estimates. As a result, group-level conclusions are more stable and less influenced by individual variability. In contrast to conventional second-level GLM approaches, PEB requires estimation of only a single full model per participant, with alternative hypotheses efficiently evaluated using Bayesian Model Reduction (Frässle & Stephan, 2022; Friston et al., 2016; Zeidman et al., 2019a; Zeidman et al., 2019b).

#### Volume of interest selection and time series extraction

**Figure 3:**
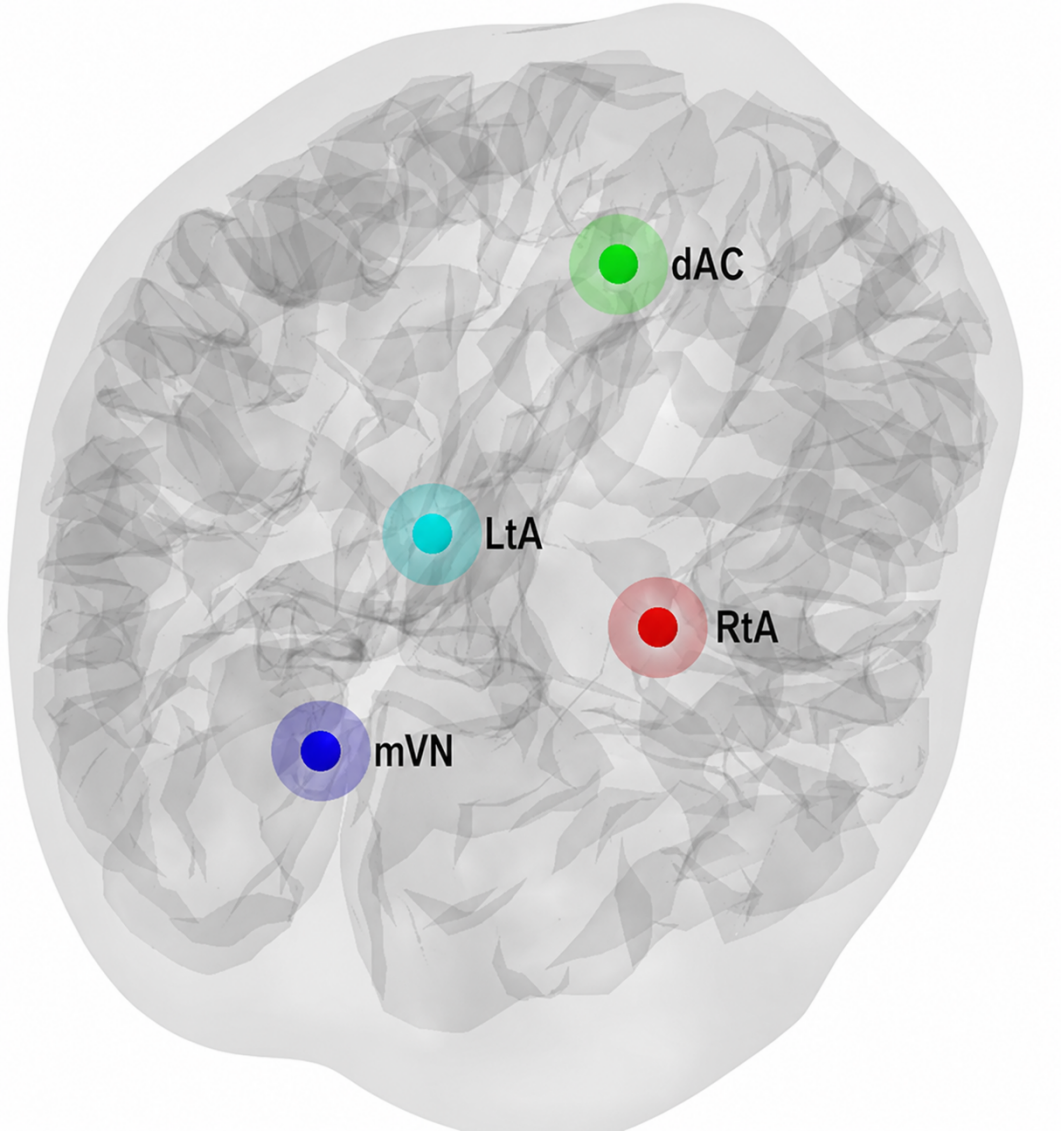
VOI locations are shown on an MNI standard brain template. Abbreviations: mVN, medial Visual Network (blue); dACC, dorsal anterior cingulate cortex (green); RtA, right amygdala (red); LtA, left amygdala (cyan).

The first step in conducting a DCM analysis involves the selection of specific brain regions and their possible connections. These regions were identified based on group-level GLM results and relevant neuroimaging literature regarding emotional processing. The Brodmann (BA) area 17, also known as the calcarine cortex, was identified as one of the volumes of interest (VOIs) corresponding to the medial Visual Network (mVN) in CONN functional analysis. Given that the seed of mVN functionally activates both the left and right anterior cingulate cortex, these two regions were selected as the combined volume of interest. Additional regions of interest included the right amygdala and the left amygdala. The inclusion of these regions is supported by extensive prior research that consistently emphasizes the critical role of the amygdala within the emotional limbic system (Heinbockel & Heinbockel, 2024; Lee et al., 2025; Lu et al., 2024). The illustration of the VOIs is presented in Fig. 3, with tabulated coordinates provided in Table 2.

Initially, two VOIs comprising white matter (WM) and cerebrospinal fluid (CSF) were defined for each participant. The VOIs were determined based on specific sphere centre coordinates: 0, −17, 39 for WM and 0, −40, −5 for CSF, each utilizing a six mm radius. These volumes were incorporated as multiple regressors in the first-level analysis, which included two valence conditions. Subsequently, a participant-specific peak was identified within the corresponding anatomical masks for each participant and VOI. These masks were constructed from BA17 and BA18 for the mVN, as well as the left and right anterior cingulum, specifically targeting the dorsal part of the anterior cingulate cortex. Additionally, predefined masks for each left and right amygdala were included.

Next, the first eigenvariate time series was extracted from the preprocessed functional data using an eight mm-radius sphere centered on each subject-specific peak. The extracted time series were adjusted for effects of interest (EOI) by regressing out nuisance variables. All active voxels within the sphere were included in the computation of the first eigenvariate, with no threshold applied. It is important to emphasize that the group-level coordinates served solely as initial reference points for identifying subject-specific peaks. A significant local peak was reliably identified within 10 mm of the starting coordinates for every participant and VOI. The Euclidean distances between the centers of the subject-level spheres and the initial group-level peaks were calculated and tabulated for both groups. Additional details regarding the time series extraction process are provided in Table S2.

**Table 2:** MNI coordinates for VOIs in the DCM analysis.

**Volume of interest (VOI)**	**Reference MNI coordinates (mm)**		
	**x**	**y**	**z**
mVN	2	−79	12
dACC	6	10	38
RtAmyg	21	−2	−17
LtAmyg	−20	−6	−15

**Notes.**

Abbreviation mVN medial Visual Network dACC dorsal anterior cingulate cortex RtAmyg right amygdala LtAmyg left amygdala

#### Model specification

The DCM model space was defined by specifying the complete pattern connectivity among the selected VOIs. Two identical one-state bilinear DCMs were constructed, one for positive valence and the other for negative valence, following the recommended methodologies for modelling effective connectivity during emotional processing (Friston, Harrison & Penny, 2003; Stephan et al., 2010). Each model estimated three types of parameters: (1) endogenous parameters (A-matrix), representing baseline effective connectivity across all conditions; (2) modulatory parameters (B-matrix), representing changes in effective connectivity induced by the positive or negative valence conditions; and (3) driving parameters (C-matrix), representing the direct influence of the stimuli on specific brain regions. These parameter types are consistent with previous DCM studies investigating valence-specific neural dynamics (Klasen et al., 2011).

Bidirectional connections were specified between the following regions for both positive and negative emotional valences. Specifically, connections were observed between the mVN and dACC, mVN and right amygdala (RtA myg), mVN and left amygdala (LtA myg), RtA myg and LtA myg, RtA myg and dACC, and LtA myg and dACC. These connections were selected based on results from GLM and functional connectivity results, and as justified previously, reflect the established roles of these regions in visual processing (mVN) (Wang et al., 2020), emotional regulation (amygdala), and cognitive-emotional integration (dACC) (Etkin, Egner & Kalisch, 2011) (refer to ‘Volume of interest selection and time series extraction’ for detailed justification and relevant literature). The dorsal anterior cingulate cortex (dACC) is a critical brain region for emotional processing and regulation, making it essential to include in DCM studies. The dACC is involved in conflict monitoring, error detection, and the integration of cognitive and emotional information, which are key components of emotional regulation. Its connectivity with other brain regions, such as the amygdala and prefrontal cortex, allows it to modulate emotional responses and influence decision-making processes (Dignath et al., 2020). Including the dACC in DCM helps capture its role in top-down control of emotions and its interactions with other neural networks, providing a more comprehensive understanding of emotional processing.

In addition to these extrinsic (between-region) connections, each region was assigned an inhibitory self-connection (diagonal elements of the A-matrix) to model intrinsic excitatory–inhibitory balance, consistent with DCM best practices (Frässle et al., 2021). In DCM, self-connections are intrinsic parameters that describe how strongly a brain region regulates its own activity. They are biologically modelled as inhibitory and reflect the balance between excitation and inhibition within a region. Functionally, self-connections act as a form of gain control, determining how sensitive a region is to incoming signals from other parts of the network.

To ensure stable neural dynamics, self-connections are constrained to be inhibitory and are estimated as unit-less log-scaling parameters applied to a default inhibitory value, typically −0.5 Hz. The effective self-connection strength (a) is given by the following equation:

\begin{eqnarray*}a=-0.5\cdot exp({A}_{self} ). \end{eqnarray*}

With this equation, the sign of the estimated parameter (A_self_) determines whether a region becomes more or less inhibitory relative to baseline, thereby affecting its sensitivity to inputs. Positive self-connection values indicate increased self-inhibition and reduced sensitivity, whereas negative values indicate reduced self-inhibition (disinhibition) and increased sensitivity. This interpretation provides a biologically meaningful and intuitive framework for understanding how task conditions modulate regional excitability in DCM analyses (Friston, Harrison & Penny, 2003; Stephan et al., 2010; Zeidman et al., 2019a; Zeidman et al., 2019b).

Positive and negative valence stimuli were designated driving inputs to the mVN and the bilateral amygdala. While group-level analyses did not reveal significant amygdala activation differences between valence conditions, the amygdala was included as the driving input target due to its established role in the subcortical visual pathway and rapid processing of emotional signals (Khalil et al., 2023; Sato et al., 2019). Modulatory effects of positive and negative valence were applied only to the self-connections within each region. This approach was done by previous study to enhance parameter identifiability (Ballotta et al., 2023; Zeidman et al., 2019a; Zeidman et al., 2019b). Because self-connections are inherently inhibitory, both endogenous and modulatory self-connection parameters are represented as unitless log-scaling values adjusting the default rate of −0.5 Hz, thus a higher self-connection parameter indicates increased inhibition.

To enhance model evidence and improve the interpretation of the A-matrix as average connectivity, stimulus timing input vectors were mean-centered (Zeidman et al., 2019a; Zeidman et al., 2019b). The DCMs were estimated using Variational Laplace, which optimizes model parameters by approximating the posterior distribution, balancing model complexity and fit to prevent overfitting. This initial model, including all specified connections and modulatory effects, is referred to as the “full” model.

### Parametric empirical Bayes

Connectivity parameters were first estimated individually using the “full” DCMs described above. A second-level analysis was then performed using PEB (Friston et al., 2016) to assess group-level commonalities and differences in connectivity. The PEB framework begins with the single-subject “full” DCMs, which include all specified connections. To facilitate an automated identification of which parameters could be eliminated without significantly diminishing model evidence, Bayesian model reduction (BMR) was applied (Zeidman et al., 2019b).

In both HC and nTBI groups, fixed connections (A-matrix) were modeled using 32 second-level parameters. This model included two between-subject effects (group mean and group difference) and 16 DCM parameters per group, encompassing four self-connections and twelve inter-regional connections. Modulatory inputs (B-matrix) were represented by 16 parameters, accounting for the two groups, with four DCM parameters assigned to each group, multiplied by two for valence conditions.

The final stage of the analysis involved Bayesian model averaging (BMA), which computed a weighted average of the parameters across all reduced models, with weights derived from the posterior probability associated with each model. Parameters with a posterior probability exceeding 0.95 were retained, ensuring that only the most reliable connectivity effects are reported. This approach provided a robust and parsimonious explanation of the observed data.

## Results

### Demographics and neurological results

Differences in demographic and neuropsychological characteristics between the HC (*n* = 20) and nTBI (*n* = 20) groups were examined using independent t-tests. Effect sizes were calculated using Hedges’ g to quantify the magnitude of group differences. The results are summarized in Table 3.

**Table 3:** Demographic and descriptive statistics of neurophysiological characteristics.

	**Mean** **HC (SD)**	**Mean** **nTBI (SD)**	**t-statistics**	**p** ^a^	**Effect Size** **(Hedges’ g)**
**Age** (HC: *n* = 20, nTBI: *n* = 20)	27.85 (7.73)	30.70 (11.78)	−0.904	0.372	−0.280
**Years of education** (HC: *n* = 20, nTBI: *n* = 20)	15.15 (2.01)	12.08 (2.90)	2.984	0.005^*^	0.925
**Days since injury**	–	40.60 (9.92)	–	–	–
**GCS** (median)	–	14.5	–	–	
**WASI-I** (HC: *n* = 11, nTBI: *n* = 19)					
Performance IQ (PIQ)	104.55 (10.78)	93.26 (10.45)	2.817	0.009^*^	1.038
Block Design	52.18 (9.99)	48.63 (5.05)	1.105	0.289	0.481
Matrix Reasoning	53.73 (10.02)	42.95 (12.39)	2.452	0.021^*^	0.904
**RAVLT** (HC: *n* = 11, nTBI: *n* = 19)					
Immediate memory	47.00 (7.94)	38.11 (8.80)	2.761	0.010^*^	1.018
Delayed memory	10.00 (2.76)	7.63 (3.34)	1.990	0.056	0.733
**RCFT** (HC: *n* = 11, nTBI: *n* = 19)					
Immediate recall	55.91 (7.52)	39.74 (17.69)	3.480	0.002^*^	1.058
Delayed recall	51.27 (11.48)	39.05 (16.58)	2.156	0.040^*^	0.795
**WCST** (HC: *n* = 11, nTBI: *n* = 19)	90.00 (17.88)	77.00 (21.02)	1.719	0.097	0.634
**CTMT** (HC: *n* = 11, nTBI: *n* = 19)	39.55 (10.84)	32.26 (14.74)	1.426	0.165	0.526

**Notes.**

Abbreviation WASI Wechsler Abbreviated Scale of Intelligence RAVLT Rey Auditory Verbal Learning Test RCFT Rey Complex Figure Test CTMT Comprehensive Trail-Making Test WCST Wisconsin Card Sorting Test HC healthy control TBI traumatic brain injury

a mean *t*-test at *p* < 0.05; Hedges’ g = 0.15, 0.40, and 0.75 are interpreted as small, medium, and large effects, respectively (Brydges, 2019).

No significant differences were found between the groups in terms of age (t(38) = −0.904, *p* = 0.372, Hedges’ g = − 0.28). However, the HC group exhibited a significantly greater number of years of formal education than the nTBI group (t(38) = 2.984, *p* = 0.005, Hedges’ g = 0.925). Within the nTBI group, the mean number of post-injury days at the time of assessment was 40.60 (SD = 9.92), and the median GCS score was 14.5. Additionally, all participants were Malay males, ensuring homogeneity with respect to ethnicity and gender.

A subset of participants (11 HC, 19 nTBI) completed a neuropsychological assessment, revealing group differences on several measures. The performance IQ (PIQ) on the WASI-I was significantly lower in the nTBI group compared to the HC group (t(28) = 2.817, *p* = 0.009, Hedges’ g = 1.038). Similarly, performance on the Matrix Reasoning subtest of the WASI-I was significantly poorer for the nTBI group (t(28) = 2.452, *p* = 0.021, Hedges’ g = 0.904), while no significant difference was observed in the Block Design subtest (t(28) = 1.105, *p* = 0.289, Hedges’ g = 0.481).

In the RAVLT, the nTBI group showed significantly impaired immediate recall (t(28) = 2.761, *p* = 0.010, Hedges’ g = 1.018) and a trend towards impaired delayed recall (*t* (28) = 1.990, *p* = 0.056, Hedges’ g = 0.733). For the RCFT, the nTBI group performed significantly worse than the HC group on both immediate recall (t(28) = 3.480, *p* = 0.002, Hedges’ g = 1.058) and delayed recall (t(28) = 2.156, *p* = 0.040, Hedges’ g = 0.795). No significant group differences were observed on the WCST (t(28) = 1.719, *p* = 0.097, Hedges’ g = 0.634) or CTMT (t(28) = 1.426, *p* = 0.165, Hedges’ g = 0.526).

These results indicate that the nTBI group exhibited impairments in general intellectual functioning (as evidenced by PIQ), fluid intelligence (as assessed by Matrix Reasoning), and both verbal and visual memory (as measured by the RAVLT and RCFT immediate and delayed recall), relative to healthy controls. While there were trends towards poorer performance on measures of executive function (WCST) and delayed verbal recall (RAVLT), these differences did not reach statistical significance.

### Brain activation

A whole-brain GLM analysis was conducted to examine differences in brain activation between the HC and nTBI groups across four emotional conditions: sad, happy, fear, and calm. The results were thresholded at a significance level of *p* < 0.05, with FWE correction applied for multiple comparisons at both the peak and cluster levels. Significant brain activations identified in this analysis are summarized in Table S1.

#### Main effect of group

**Figure 4:**
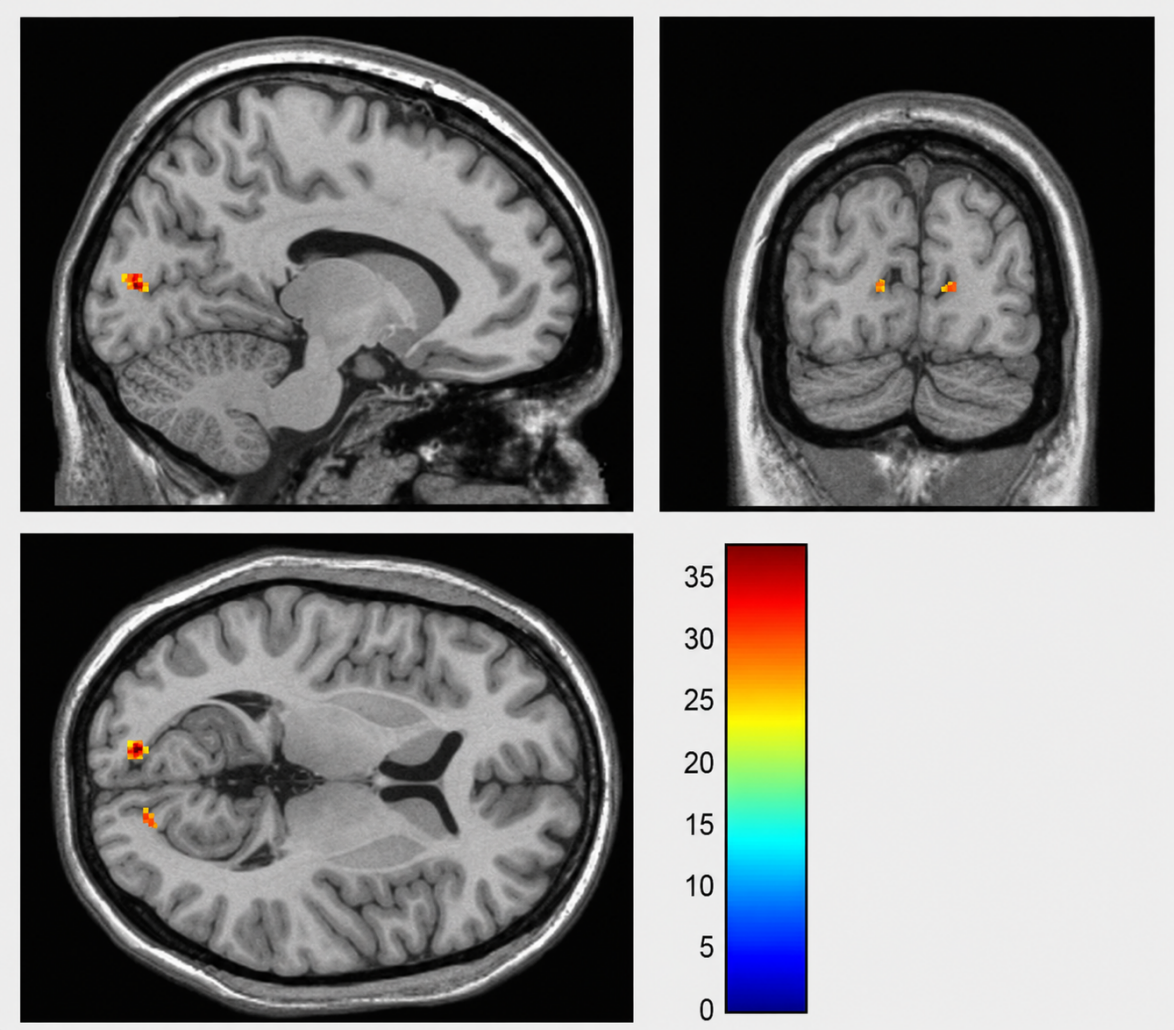
The main effect of the group showed significantly greater activation for HC > nTBI in the bilateral calcarine cortex. Thresholded at *p* < 0.05, FWE-corrected at the peak and cluster levels. Activations are displayed on sagittal (top left), coronal (top right), and axial (bottom left) views of an MNI standard brain template.

The analysis revealed a significant main effect of the group, whereby the HC group exhibited greater activation than the nTBI group in several visual processing regions. Notable areas of activation included the calcarine cortex (BA17; MNI: −9, −88, 8, Hedges’ g = 1.94), the right lingual gyrus (MNI: 13, −83, 3, Hedges’ g = 1.76), and the right calcarine cortex (MNI: 16, −83, 8, Hedges’ g = 1.72) (see Fig. 4). These findings suggest a reduced engagement of early visual processing regions in the nTBI group.

#### Main effect of emotional condition

A significant main effect of emotional condition was observed across multiple brain regions (see Fig. S1). This included bilateral activation of the inferior temporal gyrus (Left: MNI: −49, −61, −7, Hedges’ g = 1.49; Right: MNI: 51, −61, −1, Hedges’ g = 1.31), regions involved in visual object recognition and emotional processing. Additionally, changes were observed in the bilateral paracentral lobule (BA6; Left: MNI: −4, −28, 62, Hedges’ g = 1.23; Right: MNI: 4, −28, 65, Hedges’ g = 1.13) and the supplementary motor area (MNI: 4, −25, 59, Hedges’ g = 1.07), potentially indicating sensorimotor responses and motor preparation related to emotional stimuli. Detailed condition-specific activation maps and full cluster statistics are provided in Fig. S1 and Table S1.

#### Contrast analysis

A further analysis examined group differences in brain activation for each emotion condition. Across all emotional conditions, the contrast comparing HC > nTBI revealed significant activations in visual processing regions, including the left calcarine cortex (MNI: −9, −88, 8, Hedges’ g = 0.78), the right lingual gyrus (MNI: 13, −81, −4, Hedges’ g = 0.76), and the right calcarine cortex (BA17) (MNI: 16, −83, 8, Hedges’ g = 0.78). These results suggest a general reduction in early visual processing activity in the nTBI group compared to the HC group, regardless of emotional content.

When examining specific emotional conditions, the Fear HC >Fear nTBI contrast revealed a significant activation difference in the left calcarine cortex (MNI: −11, −86, 8, Hedges’ g = 0.73). The Happy HC > Happy TBI contrast showed significant activation differences in both the left calcarine cortex (MNI: −9, −88, 8, Hedges’ g = 0.78) and right calcarine cortex (BA17) (MNI: 18, −83, 8, Hedges’ g = 0.70). No significant activations were observed for the TBI > HC contrast across any of the emotional conditions, nor were there significant group differences in the sad or calm conditions.

These findings suggest that while there is a generalized reduction in visual processing activity in the nTBI group across emotional stimuli, this difference is particularly pronounced during the processing of fear and happy stimuli. The absence of significant differences in the sad and calm conditions may indicate nuanced effects of nTBI on neural responses that depend on the emotional valence and/or arousal associated with the stimuli

### Functional connectivity in healthy control and traumatic brain injury group

This study aims to examine the differences in FC between HC and nTBI participants. We employed both seed-based and ROI-based FC analyses using the CONN Toolbox. The seed-based (seed-to-ROI) analysis examined the connectivity between a predefined seed region and all other ROIs across the entire brain, identifying regions with significant correlations to the activity of the seed regions. In contrast, the ROI-based (ROI-to-ROI) analysis focused on connectivity within and between predefined ROIs, providing a broader view of network interactions among these regions. However, no significant differences were identified in the ROI-based analyses.

Based on the significant group differences observed in the calcarine cortex during the GLM analysis of brain activation (see ‘Brain activation’), the mVN (seed coordinates: 2, −79, 12) was selected as a seed region for the subsequent seed-based FC analysis. The mVN, encompassing BA17 and the calcarine cortex, is recognized as a critical hub for early-stage visual processing and is known to be affected by TBI (Amgalan et al., 2022; Ji et al., 2023; Tinney et al., 2024; Zhou et al., 2012), as supported by recent literature.

#### Across emotional condition connectivity for HC >nTBI

The seed-based analysis revealed significantly greater connectivity between mVN and several brain regions in the HC group compared to nTBI group (HC > nTBI) across all emotional conditions (see Table S3 and Fig. S2). Notable regions exhibiting such differences included the right frontal pole (Rt FP; *F* = 7.48, p_FDR_ = 0.042) and clusters within the anterior division of the cingulate cortex (ACC; *F* = 6.32, p_FDR_ = 0.042 and *F* = 9.92, p_FDR_ = 0.042) (see Fig. S2). These findings suggest a generally stronger FC between visual processing areas and higher-order cognitive and emotional control regions in healthy participants.

#### Emotional condition-specific connectivity for nTBI >HC

The analysis of emotional condition-specific effects revealed increased in FC within the nTBI group compared to the HC group across several regions during both sad and fear conditions (see Table S3 and Figs. S3–S4). Specifically, during the sad condition, the nTBI group exhibited significantly greater connectivity between the mVN and ACC (*T* = 5.55, p_FDR_ < 0.001, Hedges’ g = 0.205). Additionally, enhanced connectivity was observed between mVN and the right posterior division of the supramarginal gyrus (Rt pSMG: *T* = 5.23, p_FDR_ < 0.001, Hedges’ g = 0.119), the left supramarginal gyrus (Lt SMG; *T* = 4.71, p_FDR_ < 0.001 = 0.013, Hedges’ g = 0.159), and the Rt FP (*T* = 4.46, p_FDR_ = 0.017, Hedges’ g = 0.178) (see Fig. S3).

During the fear condition, the nTBI group also demonstrated significantly greater connectivity between the mVN and Lt SMG (*T* = 4.89, p_FDR_ = 0.031, Hedges’ g = 0.066) (see Fig. S4) Importantly, no significant connectivity differences between groups were observed during the happy or calm conditions. These findings suggest that the HC group generally exhibits stronger connectivity between the visual network and regions associated with cognitive control and emotional regulation, specifically the frontal pole and ACC. The nTBI group demonstrates increased connectivity between the visual network and regions associated with somatosensory processing and potentially emotional responses (SMG and ACC during the sad condition), particularly when processing negative emotional stimuli. The increased connectivity in the frontal pole is also observed in the nTBI group while engaged in processing these negative emotional stimuli.

### Correlation between neuropsychological performance and functional connectivity

To explore potential relationships between FC and general cognitive performance, exploratory correlation analyses were conducted using FC values obtained from seed-based analysis with the mVN as the seed. These values were correlated with neuropsychological test scores in both the HC and nTBI groups. These analyses were performed to provide contextual information regarding brain–behaviour relationships and were not part of the primary study objective, which focused on neural processing of emotional stimuli. The findings are summarized in Table S4, and scatterplots illustrating these relationships are presented in Figs. S8–S16.

#### Healthy control group

In the Healthy control (HC) group, several significant correlations were observed. For the general intelligence measured by the WASI-I, greater connectivity between the mVN and the Rt FP during the fear condition (HC > nTBI contrast) was positively correlated with both performance IQ (*r* = 0.644, *p* = 0.033) and Block Design scores (*r* = 0.750, *p* = 0.008). This finding suggests that stronger connectivity between visual processing areas and the prefrontal cortex is associated with better general intellectual functioning skills in healthy participants. For visual memory, increased connectivity between mVN and the Rt FP during the fear condition (HC > nTBI contrast) exhibited a negative correlation with RCFT Immediate Recall scores (*r* = −0.741, *p* = 0.009), whereby heightened connectivity reflects an increased cognitive effort during encoding in participants with lower immediate visual recall. Regarding executive function as measured by the WCST, greater FC between the mVN and Lt ACC was positively correlated with WCST performance during both the sad condition (*r* = 0.639, *p* = 0.034) and happy condition (*r* = 0.741, *p* = 0.009) (both comparisons reflecting HC > TBI contrasts). These results highlight the role of ACC connectivity in facilitating cognitive flexibility and executive control.

#### nTBI group

In the nTBI group, significant correlations were observed predominantly during the processing of negative emotional stimuli. Specifically, for verbal memory as measured by RAVLT, greater FC between the mVN and Lt SMG during the fear condition (nTBI > HC contrast) was negatively correlated with RAVLT Immediate Recall scores (*r* = −0.530, *p* = 0.020). This finding suggests that increased connectivity within this network is indicative of less efficient verbal memory encoding. Conversely, during the sad condition (nTBI > HC contrast), greater connectivity between the mVN and the Lt ACC was positively correlated with RAVLT Delayed Recall scores (*r* = 0.464, *p* = 0.045). For visual memory as measured by RCFT, greater FC between the mVN and the Rt FP during the sad condition (nTBI > HC contrast) was positively correlated with both RCFT Immediate Recall scores (*r* = 0.486, *p* = 0.035) and Delayed Recall scores (*r* = 0.502, *p* = 0.028), potentially indicating a compensatory role for prefrontal connectivity in supporting visual memory function after nTBI.

These findings highlight the complex relationship between FC and cognitive performance in both healthy participants and those with nTBI. In healthy controls, stronger connectivity within networks involving the prefrontal cortex and ACC generally correlated with better cognitive performance except for visual memory. In contrast, in the nTBI group, increased connectivity, particularly during negative emotional processing, was associated with poorer performance (*e.g.*, RAVLT Immediate Recall and mVN-Lt SMG connectivity during fear), suggesting potential maladaptive plasticity or inefficient compensatory mechanisms. However, increased connectivity between mVN and RtFP was associated with better visual memory, and increased mVN-Lt ACC connectivity was associated with better delayed verbal recall, possibly indicating some adaptive compensatory mechanisms.

### Dynamic causal modelling analysis results

A DCM was performed to examine effective connectivity (EC) within a network of regions involved in visual and emotional processing: mVN, dACC, Rt Amyg, and Lt Amyg. Separate DCMs were estimated for positive and negative valence conditions. The analysis focused on comparing both intrinsic (self-connections) and extrinsic (between-region) connectivity parameters between HC and nTBI groups. See Table S5 for the results of endogenous connectivity (A-matrix), Table S6 for modulatory effects (B-matrix), and Figs. S5 and S6 for visualizations of effective connectivity networks.

#### Intrinsic connectivity (self-connections)

Self-connections, which represent the intrinsic inhibitory control within each region, were generally negative in both groups, indicating self-inhibition. However, the nTBI group had significantly stronger self-inhibition in the mVN, dACC and Rt Amyg compared to the HC group (see Table S5 for the specific parameter values). This suggests reduced excitability and potentially impaired information processing within these regions in the nTBI group.

#### Extrinsic connectivity (between-region connections)

Analysis of inter-regional connectivity revealed several key differences between the HC and nTBI groups. In the HC group, the mVN exhibited a weak or negligible influence on the dACC, Rt Amyg, and Lt Amyg. In contrast, in the nTBI group, the mVN showed an increased excitatory influence on both the Rt Amyg and Lt Amyg, suggesting increased emotional processing in response to visual stimuli following injury.

In the HC group, the dACC exerted a positive (excitatory) influence on both Rt Amyg and Lt Amyg, which aligns with its role in emotion regulation. However, in the nTBI group, these connections shifted to become inhibitory (negative), suggesting a loss of top-down regulatory control from the dACC that may contribute to emotion dysregulation. Additionally, in the HC group, the Rt Amyg showed mild inhibitory connections to both the dACC and Lt Amyg. However, these connections from the Rt Amyg to the dACC did not reach statistical significance in the nTBI group, indicating a non-significant effect.

Furthermore, the Rt Amyg exerted a stronger inhibitory influence on the mVN connectivity in nTBI group, which may reflect excessive emotional modulation that interferes with visual information processing. The Rt Amyg also showed an excitatory influence on the Lt Amyg in the nTBI group, suggesting increased inter-amygdala communication following injury. In contrast, the Lt Amyg showed a strong inhibitory connection toward mVN in the HC group, while this connection became excitatory in the nTBI group. This change suggests that the Lt Amyg may increase its excitatory influence on visual processing, potentially leading to hyperactivation of emotional visual stimuli.

Moreover, the connectivity from Lt Amyg to dACC increased in both the HC and nTBI groups. Notably, the nTBI group exhibited a slightly higher connectivity, indicating heightened interaction between the amygdala and the cingulate cortex. This may reflect compensatory mechanisms for impaired emotion regulation following injury. However, the connectivity between the Lt Amyg and Rt Amyg remained inhibitory in both groups. The slightly stronger inhibition observed in nTBI group suggests a continued functional asymmetry in amygdala interactions.

#### Modulatory effects of emotional valence (B-matrix)

To investigate how emotional valence modulated intrinsic inhibitory control within each region, we examined the modulatory effects (diagonal elements of the B-matrix) in both the HC and nTBI groups (see Table S6 for full results). These parameters represent the change in self-inhibition induced by positive and negative emotional stimuli.

##### Healthy control.

In the HC group, distinct patterns of valence-dependent modulation were observed. Positive valence resulted in stronger self-inhibition in the mVN (−1.290) compared to negative valence (−0.116). This result suggests that positive emotional stimuli enhance intrinsic suppression in visual processing, potentially minimising excessive distraction from emotional content. Conversely, negative valence induced stronger self-inhibition in the dACC (−1.204) compared to positive valence (−0.570). This suggests that negative emotions increase top-down regulatory of inhibitory control, likely facilitating the effective management of affective responses. The Rt Amyg showed stronger self-inhibition during negative valence (−1.302) than positive valence (−0.603). This indicates a greater ability for emotional regulation when faced with negative stimuli, helping to maintain emotional stability. In contrast, the Lt Amyg exhibited a more balanced level of self-inhibition for both positive (−0.531) and negative (−0.459) valence. This suggests a more stable intrinsic regulation of activity in the Lt Amyg regardless of the emotional context.

##### Non-severe traumatic brain injury.

The nTBI group exhibited altered patterns of valence-dependent modulation compared to HC. In the mVN, positive valence reduced self-inhibition (0.589) compared to negative valence (−0.350). This indicates a potential overactivation of visual processing in response to positive stimuli, which could result in increased distraction or hyperactivity. Meanwhile, the dACC node showed significantly stronger self-inhibition under both valences, with more pronounced inhibition for negative valence (−2.435) compared to positive valence (−1.710). This suggests a compensatory increase in cognitive control during negative emotional processing, which may reflect an excessive effort to regulate emotions post-injury. The Rt Amyg, in contrast to HC, demonstrated a disinhibitory effect with positive valence in nTBI (0.594), while negative valence resulted in excitatory or enhanced responses (1.938) post-injury, which may lead to heightened emotional responses and challenges in suppressing emotional arousal. Similarly, a deviation from HC was observed in the Lt Amyg, where positive valence contributed to an increase in excitatory modulation (0.634), and negative valence showed a slight reduction in inhibition (0.205). This result suggests that positive emotions may amplify Lt Amyg activity following nTBI, potentially contributing to heightened responsiveness to positive stimuli.

### Parametric empirical bayes result

**Figure 5:**
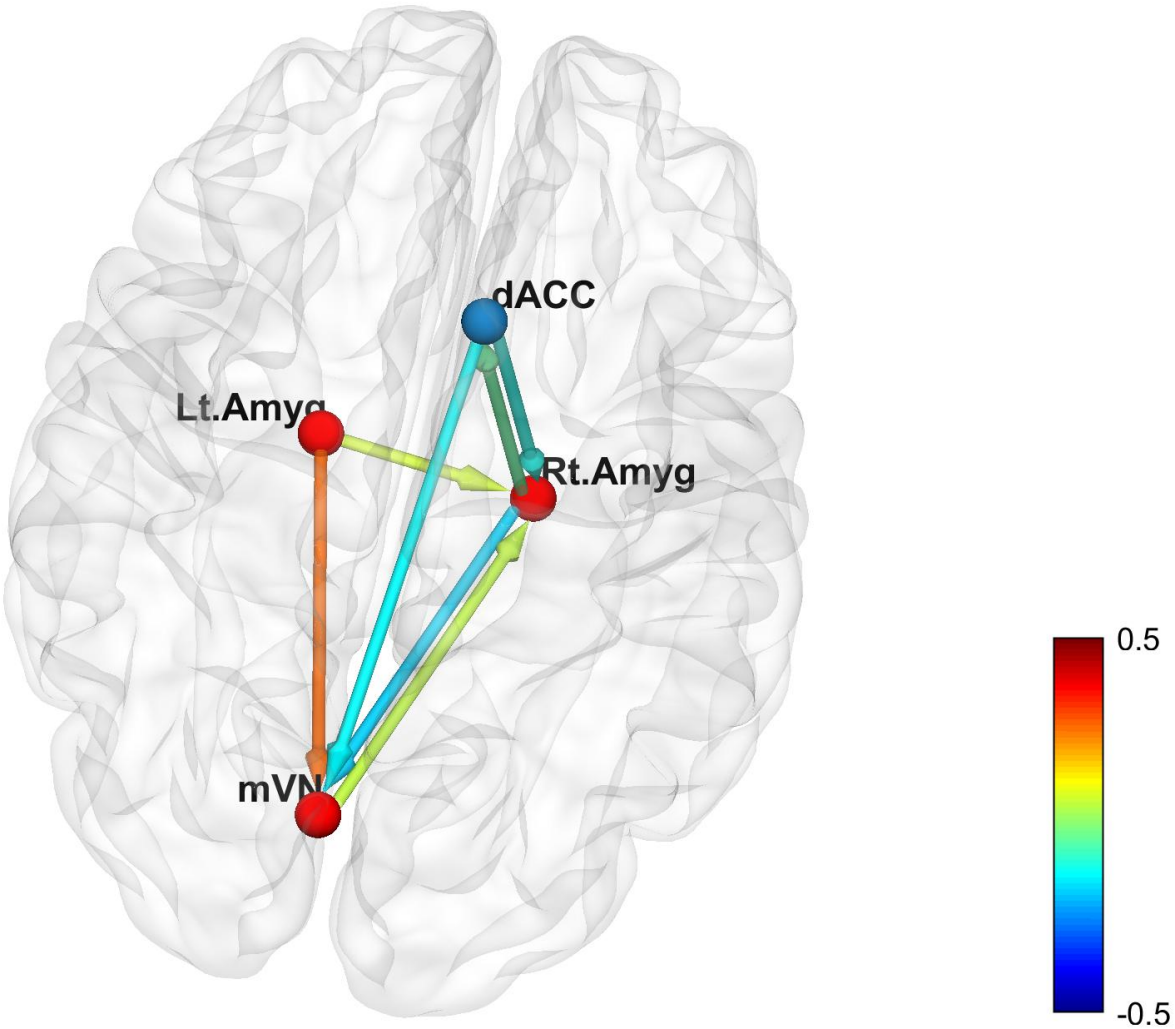
Visualization of the between-group difference (HC *vs.* nTBI) in effective connectivity or endogenous connectivity strength estimated by Parameter Empirical Bayes (PEB). Connections shown represent parameters with a posterior probability > 0.95 (95%) of being different from zero. Red and blue nodes represent the volumes of interest included in the model. Red edges indicate connections that are stronger in the nTBI group compared to the HC group; blue edges indicate connections that are stronger in the HC group. See Table 9 for parameter values.

A PEB analysis was conducted to compare the effective connectivity differences between the healthy control (HC) group and the TBI group. The analysis identified connections that were significantly stronger in either HC (negative values) or TBI (positive values). Only connections with a posterior probability of being nonzero > 0.95 were included. The difference in effective connectivity value can be seen in Table S7. For better illustration, effective connectivity difference between group is displayed in Fig. S7 and Fig. 5. Please note that the hot edges colour indicates that connections are stronger toward TBI group and while cold edges colour means that the connection is stronger toward HC group.

The results indicate that HC individuals exhibited stronger connectivity from the dACC to both mVN (−0.142) and the Rt Amyg (−0.118), suggesting that healthy individuals have greater top-down regulatory control over both visual and emotional processing. Additionally, the Rt Amyg exhibited stronger inhibitory control over mVN (−0.175) and dACC (−0.047) in HC, further supporting the notion of greater emotional regulation in the healthy brain. These findings suggest that HC individuals rely on stronger cognitive-emotional regulatory pathways to modulate emotional response and visual processing.

Conversely, the TBI group demonstrated stronger bottom-up connectivity from the Lt Amyg to mVN (0.275) and from mVN to the Rt Amyg (0.060), indicating enhanced amygdala-driven modulation of visual processing in TBI patients. Additionally, inter-amygdala connectivity (Lt Amyg to Rt Amyg) was stronger in TBI (0.072), suggesting increased bilateral amygdala communication. These findings suggest that TBI patients exhibit heightened emotional responses and reduced top-down inhibitory control, potentially contributing to difficulties in emotional regulation and attentional biases toward emotionally salient stimuli. The increased bottom-up influence from amygdala to visual processing regions may reflect excessive engagement with emotional stimuli, further disrupting cognitive-emotional balance in TBI patients.

In detailing out the modulatory difference of effective connectivity between groups upon DCM node (diagonal B-matrix), comparison between valences type were made and tabulated in Table S8. The results revealed significant modulatory differences in self-connections between the TBI and HC groups, particularly in the amygdala. The Rt Amyg exhibited stronger modulation in TBI under both positive (0.26) and negative valence (0.42), suggesting that emotional stimuli exert a greater influence on its intrinsic activity post-injury. The heightened modulation under negative valence indicates an increased sensitivity to negative emotional stimuli in TBI patients, which aligns with clinical observations of heightened emotional distress, reduced affect regulation, and increased susceptibility to negative mood states. This suggests that TBI individuals may experience greater emotional response, making it more challenging to regulate negative affect efficiently.

Similarly, the Lt Amyg showed stronger modulation in TBI, but only under positive valence (0.39), with no significant group differences in negative valence modulation. This indicates that positive emotional stimuli exert greater influence on intrinsic Lt Amyg activity in TBI individuals, possibly reflecting a shift in emotional bias toward positive stimuli as a compensatory mechanism for emotional instability. This could suggest that TBI patients may be more susceptible to heightened emotional responses to positive events, potentially to counterbalance difficulties in managing negative affect.

In contrast, no significant self-connection modulation differences were observed in the mVN or the dACC. This suggests that intrinsic emotional modulation differences in TBI are largely driven by amygdala dynamics rather than alterations in cognitive control (dACC) or sensory gating mechanisms (mVN). These findings highlight that TBI patients may experience emotion regulation deficits primarily at the level of subcortical emotional processing (amygdala) rather than through top-down cognitive control mechanisms. The stronger influence of emotion on the amygdala’s intrinsic activity suggests that emotional stimuli may become more dominant in shaping neural responses in TBI patients, potentially contributing to affective instability, heightened emotional response, and difficulties in suppressing affective responses.

## Discussion

This study employed a multimodal approach, including neuropsychological assessment, fMRI, functional connectivity, and DCM to examine the effect of nTBI on emotional processing. The nTBI demonstrated significant cognitive impairments compared to HC in general intelligence, verbal memory and visual-spatial memory. Furthermore, nTBI exhibited diminished activation in the calcarine cortex during emotional processing tasks and showed disrupted FC between the mVN and regions associated with cognitive-emotional regulation. Notably, there were alterations in EC, characterized by a reduction in top-down influence from the dACC on the amygdala and an increased bottom-up influence from the mVN. These findings primarily indicate disruption of neural circuitry supporting emotional stimulus processing following nTBI. Although complex relationships between altered connectivity patterns and cognitive performance were observed, it is important to note that brain–behaviour correlations were exploratory and were included to provide contextual interpretation rather than primary mechanistic evidence. The following sections elaborate on these findings within the context of current literature and broader neurobiological models of emotional network disruption following brain injury.

### Neuropsychological impairments: implications for cognitive function after nTBI

The observed neuropsychological impairments in the TBI group (see Table 3) align with previous literature that documents cognitive deficits following mTBI (Arciniegas, Held & Wagner, 2002; Bryant et al., 2023; Jak, 2024; Mavroudis et al., 2024; Del Pozzo et al., 2024; Shuanglong et al., 2024). Notably, the significantly lower PIQ scores and diminished performance on the matrix reasoning subset of the WASI-I indicate broader deficits in general intellectual functioning and fluid reasoning. These cognitive abilities are essential for effective problem-solving, abstract reasoning, abstract thinking, and adapting to new situations (Cattell, 1943; Irby & Floyd, 2013; Ryan, Kreiner & Gontkovsky, 2026). Therefore, their impairment can notably affect daily life functioning.

The deficits observed in verbal memory, as measured by the RAVLT immediate recall, and in visuospatial memory, as indicated by both immediate and delayed recall scores on the RCFT, warrant special attention. Memory impairments are frequently reported as one of the most commonly reported cognitive complaints following TBI (Eyolfson et al., 2025; Lencsés et al., 2025; Mitchell et al., 2023; Ponsford et al., 2025), and our findings confirm this trend even in the subacute phase post-injury. This lack of significance may be attributed to limited statistical power within the neuropsychological assessment subset or the relatively early stage of recovery. These findings align with previous research indicating that individuals with TBI often experience challenges in executive functions, such as problem-solving and cognitive flexibility (Chevignard et al., 2023; Garcia-Barrera et al., 2019; Ringdahl et al., 2019). Consequently, future research should prioritize larger sample sizes and longitudinal follow-up to comprehensively characterize the recovery trajectory of executive function and processing speed following nTBI.

### Altered brain activation and functional connectivity: disrupted visual processing and network communication

The fMRI results indicated significant changes in brain activation and functional connectivity within nTBI group. Notably, reduced activation in the calcarine cortex and lingual gyrus during the processing of emotional stimulus (see Fig. 4) suggests a critical disruption in early visual processing. The calcarine cortex (BA17), recognized as the primary visual cortex, is essential for the initial processing of visual stimuli. Damage or dysfunction in this area can result in fundamental visual perceptual deficits. The lingual gyrus is vital in more complex visual processing tasks, including object recognition, interpretation of complex visual scenes, facial expressions and emotional cues (Conway, 2018; Schettino, Gundlach & Müller, 2019). Furthermore, the inferior temporal regions are also involved in higher-level visual processing and are crucial for recognizing emotions conveyed through visual cues (Estudillo, 2025; Hu et al., 2025; Vanderlip et al., 2025). This aligns with existing literature indicating that TBI can lead to significant disruptions in deficits in visual processing pathways. Research has shown that damage to these areas can impair the processing of visual information, which is crucial for interpreting emotionally salient stimuli (Albert et al., 2022; Frankowski et al., 2021; Rauchman et al., 2023; Yue et al., 2023). Therefore, reduced activation in the calcarine cortex, lingual gyrus, and inferior temporal regions indicates that individuals with nTBI may struggle to process the visual nuances of emotional stimuli, greatly hindering their ability to perceive and interpret emotional cues accurately.

Additionally, the main effect of condition (see Fig. S1) demonstrated activations in regions commonly associated with emotional processing, including the bilateral inferior temporal gyrus, paracentral lobule, and supplementary motor area. Independent of group differences, higher-order visual and motor areas are involved in processing emotional stimuli. The paracentral lobule (BA6), part of the motor cortex, is implicated in motor planning and execution (Manyukhina et al., 2025) and may be engaged during preparing emotional responses. The supramarginal gyrus also plays a significant role, being associated with empathy and the ability to overcome emotional egocentricity bias, suggesting its involvement in understanding and responding to others’ emotions (Lei et al., 2024; Oliva et al., 2025; Silani et al., 2013). In the HC group, the activations confirm that the emotional paradigm employed successfully elicited responses. However, in the nTBI group, specific contrasts for fear and happy conditions revealed reduced activation in primary visual areas. Other researchers have only reported the impairment of negative emotion recognition such as fear was found in TBI (Murphy et al., 2022). This diminished activation may impair the ability to effectively process emotionally salient visual stimuli, leading to emotion recognition and social interaction challenges (Nahi et al., 2022; Wauters & Marquardt, 2019). These findings provide neurophysiological evidence of altered emotional processing networks following nTBI, particularly in early visual and sensorimotor integration regions.

Further insights emerged from the functional connectivity analysis using the mVN as the seed region, which revealed additional evidence of network-level disruptions (Figs. S2, S3, S4 and Table S3). The analysis showed reduced connectivity between the mVN and the Rt FP as well as the ACC in the nTBI group across all conditions (Fig. S2). This finding indicates a breakdown in communication between visual processing areas and regions essential for cognitive control and emotional regulation. The Rt FP is involved in advanced cognitive functions, including planning, decision-making, and goal-directed behaviour (Rolls, 2020). Conversely, the ACC is essential for conflict monitoring, error detection, and integrating cognitive and emotional information (Cole et al., 2024; Sönmez et al., 2025). The stronger connectivity observed in the HC group likely reflects the efficient integration of visual information with cognitive and emotional processes, facilitating appropriate behavioural responses. In contrast, the reduced connectivity seen in the nTBI group suggests that this integration is compromised, which may contribute to both cognitive and emotional challenges.

### Emotional condition-specific functional connectivity and correlations

The condition-specific functional connectivity analyses revealed that the alterations in brain connectivity were particularly pronounced during negative emotional stimuli (sad and fear) processing. During the sad condition, the nTBI group showed increased connectivity between the mVN and ACC, bilateral SMG, and Rt FP (Fig. S3). During the fear condition, increased connectivity between the mVN and Lt SMG was observed in the nTBI group (Fig. S4).

The increased connectivity between the mVN and ACC during sad stimuli processing may indicate increased recruitment of regulatory networks involved in emotional processing. This interpretation aligns with the established role of the ACC in emotion regulation and cognitive control (Chen et al., 2025; Sönmez et al., 2025; Zaid et al., 2025). Increased connectivity in this area may suggest increased recruitment of regulatory networks during emotional processing following brain injury. For example, a study on veterans with mild TBI found significant hyperconnectivity in the ACC, potentially indicating enhanced top-down control of attention networks to compensate for microstructural damage (Sheth et al., 2021).

Conversely, reduced connectivity between these regions across various conditions raises the possibility that the heightened emotional condition-specific connectivity might also represent reduced neural efficiency or altered network organisation. SMG plays a central role in sensory integration, language processing, and social cognition (Khorev et al., 2025; Oliva et al., 2025; Silani et al., 2013). In the context of nTBI, increased connectivity between the mVN and SMG during negative emotional processing, particularly under fear conditions, may indicate greater reliance on multisensory integration networks following injury (Yang et al., 2021).

Moreover, SMG is involved in multisensory integration and social-emotional processing. Therefore, the increased connectivity observed may reflect context-dependent network reorganization during fear-related emotional processing. This is consistent with research indicating that enhanced connectivity between the Amyg and SMG is associated with altered emotional processing (Timmers et al., 2022; Wood, Booth & Ko, 2024; Zhang et al., 2016).

These findings highlight significant alterations in connectivity associated with nTBI, particularly in cognitive-emotional interactions that may contribute to emotional dysregulation. Increased connectivity observed across several may reflect adaptive network recruitment in some contexts but reduced neural efficiency in others. Interpretation of connectivity increases was based on behavioural context and network organisation. However, these interpretations remain inferential and should be considered within the exploratory nature of the brain–behaviour analyses. Further research is needed to determine whether these connectivity changes represent beneficial adaptation or contribute to emotional challenges following TBI.

Exploratory correlation analysis (Table S4, Figs. S8–S16) was conducted to provide further insights into the relationship between these connectivity changes and general cognitive performance. These analyses were not part of the primary study objective, which focused on neural network alterations underlying emotional stimulus processing and should therefore be interpreted cautiously.

Notably, in the HC group, stronger connectivity in the prefrontal areas and ACC-related networks was associated with better cognitive outcomes, consistent with their known roles in executive function and cognitive control (Chen et al., 2025; Hallenbeck et al., 2025; Zhou & Geng, 2025). Conversely, relationships between connectivity and cognitive performance in the nTBI group appeared more complex and context-dependent. For example, increased FC between mVN and Lt SMG during fear processing was associated with poorer performance on the RAVLT immediate verbal recall, which may reflect reduced neural efficiency within language-related perception and processing networks, consistent with the existing literature (Moradi et al., 2017).

Interestingly, lower FC between the mVN and ACC was associated with reduced delayed recall scores in the RAVLT. This pattern may suggest altered organisation of the verbal memory network in the nTBI group, with connectivity patterns shifted from inverse coupling observed in HC to co-activation patterns following injury. These findings are consistent with the involvement of the Lt SMG and Lt ACC in attentional control and memory processes during negative emotional processing. Previous work has highlighted the role of the SMG in social–emotional processing, particularly in response to negative stimuli, which may influence performance on memory tasks such as the RAVLT (Pugh et al., 2022). Furthermore, the ACC plays a central role in integrating emotional and cognitive processes through its connections with limbic and prefrontal regions (Stevens, Hurley & Taber, 2011). It has also been suggested that negative emotional stimuli engage neural pathways involved in conflict monitoring and cognitive control, which may influence cognitive task performance (Duggirala et al., 2022).

While the RAVLT primarily assesses verbal learning and memory (Moradi et al., 2017), emotional factors can undeniably affect performance (Hepach et al., 2011). Our findings indicate that negative emotional valence is linked with increased connectivity strength towards the Lt SMG from mVN, indicating enhanced collaboration in processing fear-related information. Moreover, the role of SMG in attentional regulation could amplify the impact of emotional stimuli on memory performance, as effective attentional control allows individuals to focus on relevant emotional cues while filtering out distractions (Menon & D’Esposito, 2021). Research even suggests that disruptions in the SMG, such as those induced by transcranial magnetic stimulation (TMS), can impair spatial attention (Schenkluhn et al., 2008). In contrast, the RCFT, as an indicator of visuo-constructional ability and visual memory, reveals a significant positive correlation between FC from mVN to frontoparietal regions during sad conditions and both immediate and delayed RCFT scores in the TBI group. Though this connection appears beneficial, nTBI participants still score significantly lower than HC, indicating persistent deficits in visual memory and cognitive control (Corbetta, 2012; Marek & Dosenbach, 2018). The negative FC observed in the HC group, where one region activates while the other deactivates, contrasts sharply with the co-activated patterns in nTBI, further complicating cognitive flexibility. The emotional distress stemming from nTBI, characterized by frustration, anxiety, or depression, certainly impacts cognitive abilities, amplifying deficits in attention and memory that hinder performance on tasks like the RCFT (Lydon-Staley et al., 2019).

Together, these findings help contextualise how alterations in connectivity in nTBI may be related to emotional regulation and broader cognitive functioning, emphasising the need for continued investigation of these complex relationships. However, brain–behaviour relationships observed in this study should be interpreted cautiously, as correlation analyses were exploratory and not designed to establish causal relationships between emotional neural processing and global cognitive performance. An exploratory summary of the correlation between FC and neurophysiological performance provides additional insights. In the HC group, positive correlations between prefrontal connectivity and IQ/visuospatial performance suggest that stronger functional integration within prefrontal networks may support higher cognitive performance. Conversely, the negative correlation between FC in the Rt FP and visual memory (RCFT immediate recall) may reflect task-dependent neural resource allocation rather than simple performance enhancement. Additionally, associations between ACC connectivity and executive function performance are consistent with the established role of this region in cognitive control and adaptive regulation during emotional processing (Bounoua et al., 2025; Journée et al., 2023; Ye et al., 2025). Collectively, these observations are consistent with known functional roles of prefrontal and anterior cingulate networks in supporting intelligence, memory, and executive functions.

In contrast, findings in the nTBI group suggest more heterogeneous FC-cognition relationships across verbal and visual memory domains. For example, increased connectivity between the mVN and Lt SMG during fear processing was associated with poorer immediate verbal recall. This pattern may indicate reduced neural efficiency or altered network organisation following injury, although compensatory reorganisation cannot be excluded. These observations highlight the complexity of post-injury brain–behaviour relationships and support the need for future studies specifically designed to examine interactions between emotional regulation networks and cognitive function following nTBI.

### DCM: understanding directionality

To further explain the neural mechanisms associated with the observed changes in emotional processing and connectivity, we employed DCM to analyze the directional influences or effective connectivity within a network that includes the mVN, dACC and bilateral Amyg. Distinct models were constructed for positive and negative valence conditions. Group-level connectivity differences were then evaluated using PEB to compare connectivity parameters between HC participants and those with nTBI (Table S5–S8; Fig. 5 and Figs. S5–S7). Connectivity differences are reported using explicit directional comparisons (HC *vs* nTBI) together with the connection sign (excitatory *vs* inhibitory) to improve clarity of interpretation.

Increases in functional or effective connectivity were interpreted using predefined performance and network-organisation-based criteria. Connectivity increases were considered potentially compensatory when associated with preserved or relatively better behavioural performance, or when they reflected connectivity patterns directionally similar to those observed in the HC group. In contrast, increases were interpreted as potentially inefficient or maladaptive when associated with poorer behavioural performance, reduced network segregation, or broader non-selective cross-network coupling. This interpretation framework aligns with contemporary network models of brain injury (Hillary & Grafman, 2017; Medaglia, 2017).

### Disrupted top-down control and heightened amygdala influence

The DCM results revealed a significant alteration in network dynamics in the nTBI group compared to the HC group. In HC participants, the dACC demonstrated a positive (excitatory) influence on bilateral Amyg, consistent with its established role in top-down emotion regulation (Kim et al., 2025; Morawetz, Berboth & Bode, 2024; Wang et al., 2024b). This finding suggests that, in HC group, the dACC actively modulates Amyg activity, contributing to appropriate emotional responses. Critically, in the nTBI group, the dACC-to-Amyg connections showed reduced excitatory influence or shifted toward inhibitory coupling, indicating a loss of effective top-down regulatory control. This loss of top-down control from the dACC may significantly contribute to the emotional dysregulation commonly observed after TBI (Díaz et al., 2025; Fini & Tyler, 2020; Poggi, Klaus & Pryce, 2024; Wang et al., 2024a). These findings resonate with previous studies that have reported reduced prefrontal-Amyg connectivity in TBI correlating with emotional instability (Cole et al., 2024; Jin et al., 2024).

Furthermore, in the HC group, the mVN employed only weak influences on the Amyg. However, in the nTBI group, the mVN-to-Amyg connections demonstrated stronger excitatory influence, suggesting that visual input exerts a more direct drive on emotional processing regions following injury. This pattern indicates a relative shift toward bottom-up, stimulus-driven emotional processing, potentially bypassing normal regulatory influence from the dACC. This transition towards bottom-up, emotionally driven modulation of visual processing could contribute to heightened emotional response and attentional biases towards emotional stimuli (Amorapanth et al., 2018).

In addition to these changes, altered inter-Amyg connectivity was observed. In the nTBI group, the Rt Amyg showed increased inhibitory influence on the mVN relative to HC, potentially reflecting compensatory suppression of visual processing during heightened emotional drive. Notably, Rt Amyg–Lt Amyg connectivity was inhibitory in HC but became excitatory in nTBI, indicating increased inter-Amyg coupling that may amplify emotional signal propagation (Gimbel et al., 2021). Click or tap here to enter text.

In HC group, the Lt Amyg exerted inhibitory influence on the mVN, whereas in nTBI this connection was characterised by excitatory influence, suggesting enhanced Amyg-driven facilitation of visual processing following injury. Furthermore, Lt Amyg–dACC connectivity was stronger in nTBI than HC, indicating increased Amyg–cingulate interaction that may reflect compensatory recruitment during impaired emotion regulation. In contrast, Lt Amyg–Rt Amyg connectivity remained inhibitory in both groups, with greater inhibitory strength observed in nTBI, suggesting preserved but altered inter-Amyg regulatory balance.

Overall, these results indicate reduced top-down regulatory influence and increased bottom-up emotional drive in nTBI relative to HC.

#### Valence-specific modulation of intrinsic activity

The analysis examining the modulatory effects of emotional valence (B-matrix; Table S6) revealed distinct group differences between the HC and nTBI groups. In the HC group, positive valence was associated with increased self-inhibition in the mVN, suggesting regulation of visual input to reduce distraction from emotionally salient stimuli. In contrast, negative valence was associated with increased self-inhibition in the dACC and right Amyg, consistent with enhanced cognitive–emotional regulatory control. The left Amyg showed relatively balanced modulation across valence conditions, suggesting stable intrinsic emotional regulation in the HC group.

In contrast, the nTBI group showed altered valence-dependent modulation patterns. Positive valence was associated with reduced self-inhibition in the mVN relative to the HC group, suggesting increased visual responsiveness to positive emotional stimuli (Frankowski et al., 2021). In the nTBI group, the dACC demonstrated increased intrinsic inhibition across both valence conditions, which may reflect increased regulatory effort but potentially reduced efficiency of cognitive control mechanisms.

Furthermore, the right Amyg showed reduced inhibitory regulation during positive valence and increased excitatory modulation during negative valence in the nTBI group relative to the HC group, indicating heightened Amyg responsiveness, particularly to negative emotional stimuli. Similarly, the left Amyg showed increased excitatory modulation during positive valence and reduced inhibitory modulation during negative valence, suggesting altered emotional gain control and increased emotional sensitivity following injury.

These findings suggest that the nTBI group demonstrates altered valence-dependent regulation of intrinsic limbic and control network activity, which may contribute to altered emotional processing following injury.

#### Implications for emotional dysregulation and cognitive deficits

The findings from the DCM analyses collectively support a neural framework consistent with the emotional and cognitive challenges reported following nTBI. Disrupted top-down regulation from the dACC and stronger bottom-up drive from the Amyg suggest a neural architecture that is more reactive to emotional stimuli, with reduced regulatory control efficiency. This network configuration may contribute to increased emotional instability, enhanced sensitivity to emotional distraction, and difficulty disengaging from emotionally salient information. These alterations are consistent with the cognitive differences observed in the present neuropsychological assessments and align with findings reported in the broader TBI literature.

### A multimodal approach on emotional processing deficits in nTBI

The findings of this study illustrate how nTBI disrupts the complex balance between cognitive control and emotional response. Each analysis conducted, including neuropsychological assessments, fMRI activation, FC evaluations, and DCM, offers valuable insights. When considered together, these findings suggest that the nTBI group shows a relative shift toward bottom-up, emotion-driven processing.

Reduced activation within early visual regions suggests potential alterations in sensory processing of emotional stimuli. Network-level connectivity changes further support this interpretation, including reduced top-down regulatory influence from the dACC and increased excitatory influence from the mVN to the Amyg. These alterations may result in emotional responses that are less effectively regulated and more strongly influenced by incoming sensory input.

Valence-specific modulatory effects further support this hypothesis, as nTBI group demonstrated increased Amyg responsiveness, particularly during negative emotional processing. Neuropsychological differences, particularly in memory and executive function, may both influence and be influenced by these neural alterations. For instance, reduced working memory capacity may limit the ability to maintain cognitive control during emotional distraction, while increased emotional reactivity may further interfere with cognitive processing.

Together, this multimodal approach suggests that interventions targeting both cognitive function and emotional regulation may be beneficial for managing the complex consequences associated with nTBI. Future work should investigate integrated therapeutic approaches to further evaluate potential treatment optimisation.

## Conclusions and limitations

This multimodal approach provides evidence consistent with nTBI, even during the subacute phase, and is associated with alterations in brain function and cognitive performance, particularly in networks supporting emotional stimulus processing. The findings emphasize the vulnerability of early visual processing networks, disruption of top-down regulatory influences originating from the dACC, and increased Amyg-driven bottom-up signalling, particularly during negative emotional processing. These neural alterations were reflected in reduced activation and altered functional and effective connectivity, suggesting a shift toward more stimulus-driven, less-regulated emotional processing following nTBI.

DCM combined with PEB provides a mechanistic framework for interpreting these findings, demonstrating reduced top-down cognitive control from the dACC to limbic regions alongside increased Amyg influence on visual processing networks. Together, these results support the hypothesis that nTBI is associated with disrupted integration between cognitive control and emotional processing systems, which may contribute to emotional dysregulation and reduced cognitive efficiency following injury.

Exploratory brain–behaviour relationships observed in this study were not part of the primary study objective, which focused on characterising neural mechanisms underlying emotional stimulus processing following nTBI. Accordingly, correlations between connectivity measures and neuropsychological performance should be interpreted as hypothesis-generating rather than confirmatory and require validation in studies specifically designed to examine affective symptom–neural coupling.

These findings highlight the importance of incorporating multimodal neuroimaging and neuropsychological evaluation when assessing functional outcomes following nTBI. These results also suggest potential intervention targets, including approaches to strengthen cognitive control, such as cognitive training, and to improve emotional regulation through cognitive rehabilitation, neurofeedback, or mindfulness-based intervention. Future work should prioritise longitudinal designs to characterise recovery trajectories and evaluate the effectiveness of targeted therapeutic approaches.

Several limitations should be considered when interpreting the findings of this study. First, the relatively small sample size (*n* = 20 per group), while sufficient for detecting large effects, may have limited statistical power to detect more stronger differences, particularly in the neuropsychological assessments and some of the condition-specific fMRI analyses. Recruitment from the Department of Emergency provided a clinically representative sample of nTBI cases but may have introduced selection bias. Furthermore, the recruitment and follow-up were affected by the COVID-19-related movement restrictions, which contributed to reduced completion of neuropsychological assessments in a subset of participants.

Second, the cross-sectional design of this study prevents causal inferences regarding the relationship between nTBI and the observed neural and cognitive alterations. Although demographic matching and exclusion of pre-existing neurological or psychiatric conditions reduced confounding risk, pre-injury neural differences cannot be fully excluded. Longitudinal studies following individuals from the acute to chronic recovery phases are essential for understanding the temporal dynamics of brain and cognitive changes after TBI.

Third, this study included only male participants. This decision was made to reduce biological variability in this mechanistic neuroimaging study and to minimise sex-related differences in emotional processing and brain connectivity. However, this limits generalisability of the findings. Future studies should include female participants to evaluate potential sex-specific neural responses to nTBI.

Fourth, detailed lesion-based stratification by location was not feasible due to clinical heterogeneity among patients. Although clinical severity classification was available, variability in injury mechanism and lesion distribution may influence connectivity patterns and should be explored in future studies incorporating structural imaging or diffusion-based lesion characterisation. To improve transparency, a supplementary table (Table S9) is provided detailing the injury locations for each participant based on clinical imaging reports. Due to heterogeneity and overlap of lesion sites, stratification by injury location was not feasible. This information is included to aid interpretation, and the limitation is acknowledged in the study.

Fifth, the fMRI paradigm employed passive viewing of static emotional images. While this approach provides controlled stimulus presentation, it lacks ecological validity compared with real-world emotional interactions. Future studies may benefit from using dynamic or interactive emotional paradigms, such as video-based or virtual reality stimuli.

Sixth, while DCM provides insight into directional neural interactions, it is a model-based approach and results depend on model specification and the selected region of interest. Future studies should combine DCM with complementary network analysis approaches, including graph theoretical metrics and whole-brain network modelling, to provide a more comprehensive characterisation of network alterations following nTBI.

Finally, correlations between connectivity measures and neuropsychological performance were exploratory and not designed to establish causal relationships between emotional neural processing and global cognitive performance. These findings should therefore be interpreted cautiously and confirmed in future studies incorporating affective symptom-specific behavioural measures.

##  Supplemental Information

10.7717/peerj.21593/supp-1 Supplemental Information 1 The main effect of emotional condition showed significant activations (*p* < 0.05, FWE-corrected at the peak and cluster levels) Activations are displayed on sagittal (top left), coronal (top right), and axial (bottom left) views of an MNI standard brain template. Significant clusters were observed in the supplementary motor area (best seen in sagittal view), supramarginal and paracentral lobule (best seen in coronal view), and the bilateral inferior temporal gyrus (best seen in the axial view)

10.7717/peerj.21593/supp-2 Supplemental Information 2 Seed-based functional connectivity (mVN seed) demonstrated greater connectivity in HC compared to nTBI participants across conditions (pFDR > 0.05 at the cluster levels) Significant increases in FC were observed in the right frontal pole and bilateral anterior division of the cingulate cortex.

10.7717/peerj.21593/supp-3 Supplemental Information 3 Seed-based functional connectivity (mVN seed) demonstrated greater connectivity in nTBI compared to HC participants during sad condition (pFDR > 0.05 at the cluster levels) Significant increases in FC were observed in the anterior division of the cingulate cortex, bilateral supramarginal gyrus and right frontal pole.

10.7717/peerj.21593/supp-4 Supplemental Information 4 Seed-based functional connectivity (mVN seed) demonstrated greater connectivity in nTBI compared to HC participants during fear condition (pFDR > 0.05 at the cluster levels). Significant increases in FC were observed in the left supramarginal gyrus

10.7717/peerj.21593/supp-5 Supplemental Information 5 Visualization of the average effective connectivity network or endogenous connectivity strength (A-matrix) for the HC group after Bayesian Parameter Averaging (BPA) Connections shown represent parameters with a posterior probability > 0.95 (95%). Red nodes represent the region where input is being set in the model.

10.7717/peerj.21593/supp-6 Supplemental Information 6 Visualization of the average effective connectivity network or endogenous connectivity strength (A-matrix) for the nTBI group after Bayesian Parameter Averaging (BPA) Connections shown represent parameters with a posterior probability > 0.95 (95%). Red nodes represent the region where input is being set in the model.

10.7717/peerj.21593/supp-7 Supplemental Information 7 The matrix representation of the difference in the average effective connectivity or endogenous connectivity strength (A-matrix) between HC and nTBI groups

10.7717/peerj.21593/supp-8 Supplemental Information 8 Functional connectivity strength from mVN to Rt FP versus Block design psychological score in HC group (fear condition). mVN, medial visual network; FP, Frontal Pole; R^2^, *p* < 0.05, Pearson Correlation

10.7717/peerj.21593/supp-9 Supplemental Information 9 Functional connectivity strength from mVN to Rt FP versus IQ psychological score in HC group (fear condition). mVN, medial visual network; FP, Frontal Pole; IQ, Performance IQ; R^2^, *p* < 0.05, Pearson Correlation

10.7717/peerj.21593/supp-10 Supplemental Information 10 Functional connectivity strength from mVN to Rt FP versus RCFT i psychological score in HC group (fear condition). mVN, medial visual network; FP, Frontal Pole; RCFT i, Rey Complex Figure Task immediate; R^2^, *p* < 0.05, Pearson Correlation

10.7717/peerj.21593/supp-11 Supplemental Information 11 Functional connectivity strength from mVN to Lt AC versus WCST psychological score in HC group (sad condition). mVN, medial visual network; AC, Anterior Cingulate; WCFT, Wisconsin Card Sorting Test; Lt, Left; R^2^, *p* < 0.05, Pearson Correlation

10.7717/peerj.21593/supp-12 Supplemental Information 12 Functional connectivity strength from mVN to Lt AC versus WCST psychological score in HC group (sad condition). mVN, medial visual network; AC, Anterior Cingulate; WCFT, Wisconsin Card Sorting Test; Lt, Left; R^2^, *p* < 0.05, Pearson Correlation

10.7717/peerj.21593/supp-13 Supplemental Information 13 Functional connectivity strength from mVN to Lt SMG versus RAVLT (immediate) psychological score in nTBI group (fear condition). mVN, medial visual network; Lt, left; SMG, supramarginal gyrus; RAVLT i, Rey Auditory Verbal Learning Test (immediate); R [sup

10.7717/peerj.21593/supp-14 Supplemental Information 14 Functional connectivity strength from mVN to Lt AC versus RAVLT (delayed) psychological score in nTBI group (sad condition). mVN, medial visual network; Lt, left; AC, anterior cingulate; RAVLT_d, Rey Auditory Verbal Learning Test (delayed); R ^2^

10.7717/peerj.21593/supp-15 Supplemental Information 15 Functional connectivity strength from mVN to Rt FP versus RCFT (immediate) psychological score in nTBI group (sad condition) (one-sided t-test). mVN, medial visual network; Rt, right; FP, Frontal pole; RCFT_i, Rey Complex Figure Test (immediate); R [sup]2

10.7717/peerj.21593/supp-16 Supplemental Information 16 Functional connectivity strength from mVN to Rt FP versus RCFT d (delayed) psychological score in nTBI group (sad condition) (one-sided t-test). mVN, medial visual network; Rt, right; FP, Frontal pole; RCFT_d, Rey Complex Figure Test (delayed); R [sup]2[s

10.7717/peerj.21593/supp-17 Supplemental Information 17 The significant brain activation comparing the HC and nTBI groups

10.7717/peerj.21593/supp-18 Supplemental Information 18 Details of VOIs extraction. For each group VOI, the table shows (MNI) coordinates, the average number of activated voxels within centre radius of the local 8 mm-sphere, and its distance to the group-level peak

10.7717/peerj.21593/supp-19 Supplemental Information 19 Functional connectivity differences between HC and nTBI groups for seed-based analysis with mVN as the seed region

10.7717/peerj.21593/supp-20 Supplemental Information 20 The correlation between seed-based FC (mVN seed) and neuropsychological performance in HC and nTBI groups

10.7717/peerj.21593/supp-21 Supplemental Information 21 Endogenous connectivity strength (A-matrix) from the DCM analysis for HC and nTBI groups

10.7717/peerj.21593/supp-22 Supplemental Information 22 The average connectivity strength of self-connection by the modulatory influence of positive and negative valence across experimental conditions (Diagonal of B-matrix) in both group

10.7717/peerj.21593/supp-23 Supplemental Information 23 Between-group differences (HC vs. nTBI) in effective connectivity or endogenous connectivity strength from PEB analysis

10.7717/peerj.21593/supp-24 Supplemental Information 24 Between-group differences (HC vs. nTBI) in modulatory effects of positive and negative valance on self-connections (B-matrix) from PEB analysis

10.7717/peerj.21593/supp-25 Supplemental Information 25 List of participants enrolled in fMRI experiment and their injury information

10.7717/peerj.21593/supp-26 Supplemental Information 26 fMRI,reaction time performance and psychological assessment score of HC vs nTBI dataset

10.7717/peerj.21593/supp-27 Supplemental Information 27 STROBE Checklist

10.7717/peerj.21593/supp-28 Supplemental Information 28 Codebook for data and code file

## Additional Information and Declarations

### Data Availability

The following information was supplied regarding data availability: The raw data is available in the Supplemental File. The raw fMRI data for nTBI effect on emotional condition study is available at Zenodo: - Omar, H., Abdul Rahman, M. R., Abd Hamid, A. I., & Abdullah, J. M. (2025). Raw fMRI data for nTBI effect on emotional condition study [Data set]. Zenodo. https://doi.org/10.5281/zenodo.15666509.
