# Supplementary material for: Investigation of brain connectivity alteration in the non-severe traumatic brain injury: an emotional condition study
Source: PeerJ. 2026 Aug 5;14:e21593. doi: 10.7717/peerj.21593 (PMC13452427; doi:10.7717/peerj.21593)
Supplement: Supplementary file 1 [file peerj-14-21593-s017.docx]

| **Condition** | **Cluster Size** | ***F* or T*** | **Z_E_** | **Aal (Brodmann) Area** | **Laterality** | **MNI Coordinate** | | |  |
| --- | --- | --- | --- | --- | --- | --- | --- | --- | --- |
|  |  |  |  |  |  | **x** | **y** | **z** | **Effect Size**  (Hedges' g) |
| Main Effect of Group* | 32 | 37.68 | 5.67 | Calcarine | Left | -9 | -88 | 8 | 1.94 |
|  | 7 | 31.00 | 5.17 | Lingual | Right | 13 | -81 | -4 | 1.76 |
|  | 7 | 29.69 | 5.07 | Calcarine (BA17) | Right | 16 | -83 | 8 | 1.72 |
| Main Effect of Condition* | 497 | 22.24 | 6.80 | Inferior Temporal (BA19) | Left | -49 | -61 | -7 | 1.49 |
|  | 461 | 18.54 | 6.21 | Inferior Temporal (BA19) | Right | 51 | -61 | -10 | 1.36 |
|  | 40 | 15.10 | 5.58 | Paracentral Lobule (BA6) | Left | -4 | -28 | 62 | 1.23 |
|  | 14 | 12.87 | 5.12 | Supra Marginal | Left | -59 | -26 | 29 | 1.13 |
|  | 2 | 12.42 | 5.02 | Paracentral Lobule (BA6) | Right | 4 | -31 | 65 | 1.11 |
|  | 1 | 11.47 | 4.80 | Supplementary Motor Area (BA6) | Right | 4 | -25 | 59 | 1.07 |
| HC>nTBI | 45 | 6.14 | 5.79 | Calcarine | Left | -9 | -88 | 8 | 0.78 |
|  | 9 | 5.57 | 5.30 | Lingual | Right | 13 | -81 | -4 | 0.75 |
|  | 12 | 5.43 | 5.20 | Calcarine (BA17) | Right | 16 | -83 | 8 | 0.74 |
| Fear HC>Fear nTBI | 7 | 5.40 | 5.15 | Calcarine | Left | -11 | -86 | 8 | 0.73 |
| Happy HC>Happy nTBI | 45 | 6.04 | 5.71 | Calcarine | Left | -9 | -88 | 8 | 0.78 |
|  | 1 | 4.94 | 4.75 | Calcarine (BA17) | Right | 18 | -83 | 8 | 0.70 |
| * indicate F-value |  |  |  |  |  |  |  |  |  |

*Table S1 The significant brain activation comparing the HC and nTBI groups.*
