# Supplementary material for: Investigation of brain connectivity alteration in the non-severe traumatic brain injury: an emotional condition study
Source: PeerJ. 2026 Aug 5;14:e21593. doi: 10.7717/peerj.21593 (PMC13452427; doi:10.7717/peerj.21593)
Supplement: Supplementary file 2 [file peerj-14-21593-s018.docx]

*Supplementary Table S2 Details of VOIs extraction. For each group VOI, the table shows (MNI) coordinates, the average number of activated voxels within centre radius of the local 8 mm-sphere, and its distance to the group-level peak.*

| **Group** | **VOIs** | | **Reference Coordinate** | | | | | | **F-Value (average)** | | **NOV (average)** | | **Average distance (mm)** | |
| --- | --- | --- | --- | --- | --- | --- | --- | --- | --- | --- | --- | --- | --- | --- |
| HC | mVN | | 2 | | -79 | | 12 | | 130.60 | | 63 | | 6.79 | |
|  | dACC | | 6 | | 10 | | 38 | | 11.84 | | 18 | | 9.78 | |
|  | RtAmyg | | 21 | | -2 | | -17 | | 24.31 | | 40 | | 4.61 | |
|  | LtAmyg | | -20 | | -6 | | -15 | | 21.00 | | 33 | | 4.24 | |
| nTBI | mVN | | 2 | | -79 | | 12 | | 71.66 | | 62 | | 7.59 | |
|  | dACC | 6 | | 10 | | 38 | | 13.08 | | 22 | | 9.23 | |  |
|  | RtAmyg | 21 | | -2 | | -17 | | 17.93 | | 23 | | 6.13 | |  |
|  | LtAmyg | -20 | | -6 | | -15 | | 16.00 | | 22 | | 3.88 | |  |

*HC,healthy control; nTBI, non-severe traumatic brain injury group; mVN , medial Visual Network; dACC, dorsal part of the Cingulate Cortex; RtAmyg, right amygdala; LtAmyg, left amygdala; NOV, number of voxel; VOI, volume of interest.*
