# Supplementary material for: Investigation of brain connectivity alteration in the non-severe traumatic brain injury: an emotional condition study
Source: PeerJ. 2026 Aug 5;14:e21593. doi: 10.7717/peerj.21593 (PMC13452427; doi:10.7717/peerj.21593)
Supplement: Supplementary file 3 [file peerj-14-21593-s019.docx]

*Table S3 Functional connectivity differences between HC and nTBI groups for seed-based analysis with mVN as the seed region.*

| **Condition** | **Activated regions** | **Coordinate (x y z)** | | | **Cluster size** | | **p^a^** | **F-* or T-**  **statistics** | **Effect size^b^** |
| --- | --- | --- | --- | --- | --- | --- | --- | --- | --- |
| HC>nTBI* | Rt FP | 32 | 46 | -22 | | 38 | 0.042 | 7.48 | - |
|  | AC (anterior division) | 6 | 10 | 38 | | 35 | 0.042 | 6.32 | - |
|  | AC (anterior division) | -8 | 20 | 28 | | 33 | 0.042 | 9.92 | - |
| Sad nTBI> Sad HC | AC (anterior division) | -4 | 8 | 40 | | 172 | 0.000 | 5.55 | 0.205 |
|  | Rt SMG (posterior division) | 66 | -34 | 30 | | 123 | 0.000 | 5.23 | 0.119 |
|  | Lt SMG | -58 | -48 | 28 | | 73 | 0.013 | 4.71 | 0.159 |
|  | Rt FP | 32 | 56 | 12 | | 65 | 0.017 | 4.46 | 0.178 |
| Fear nTBI>Fear HC | Lt SMG | -56 | -40 | 36 | | 61 | 0.031 | 4.89 | 0.066 |

Abbreviation: FP = Frontal Pole; AC = Anterior Cingulate; SMG = supramarginal gyrus; mVN = medial Visual Network (2, -79, 12) Coordinates follow the standard Montreal Neurological Institute (MNI) template in millimeters (mm). ^a^pFDR < 0.05 at cluster threshold and p*_uncorrected_* <0.001 at the voxel level ^b^Hedges’g. * indicate F-value.
