# Supplementary material for: Investigation of brain connectivity alteration in the non-severe traumatic brain injury: an emotional condition study
Source: PeerJ. 2026 Aug 5;14:e21593. doi: 10.7717/peerj.21593 (PMC13452427; doi:10.7717/peerj.21593)
Supplement: Supplementary file 4 [file peerj-14-21593-s020.docx]

*Table S4 The correlation between seed-based FC (mVN seed) and neuropsychological performance in HC and nTBI groups*

| **Group** | **Domain** | **Contrast Condition** | **ROI  (***x y z***)** | **Mean FC HC (SD)** | **Mean FC nTBI (SD)** | **R** | **^a^p** |
| --- | --- | --- | --- | --- | --- | --- | --- |
| **HC** | **WASI 1** |  |  |  |  |  |  |
|  | Block Design | HC>nTBI (Fear) | **Rt FP**  *(32 46 -22)* | 0.13 (0.22) | -0.43 (0.18) | 0.750 | 0.008 |
|  | IQ | HC>nTBI (Fear) | **Rt FP**  *(32 46 -22)* | 0.13 (0.22) | -0.43 (0.18) | 0.644 | 0.033 |
|  | **RCFT** |  |  |  |  |  |  |
|  | Immediate | HC>nTBI (Fear) | **Rt FP**  *(32 46 -22)* | 0.13 (0.22) | -0.43 (0.18) | -0.741 | 0.009 |
|  | **WCST** |  |  |  |  |  |  |
|  |  | HC>nTBI (Sad) | **Lt ACC ant**  *(-8 20 28)* | -0.18 (0.24) | 0.19 (0.27) | 0.639 | 0.034 |
|  |  | HC>nTBI (Happy) | **Lt ACC ant**  *(-8 20 28)* | 0.06 (0.17) | -0.08 (0.19) | 0.741 | 0.009 |
| **nTBI** | **RAVLT** |  |  |  |  |  |  |
|  | Immediate | nTBI>HC (Fear) | **Lt SMG ant**  *(-52 -40 36)* | -0.24 (0.20) | 0.07 (0.19) | -0.530 | 0.020 |
|  | Delayed | nTBI>HC (sad) | **Lt AC**  *(-4 8 40)* | -0.14 (0.18) | 0.20 (0.21) | 0.464 | 0.045 |
|  | **RCFT** |  |  |  |  |  |  |
|  | Immediate | nTBI>HC (sad) | **Rt FP**  *(32 56 12)* | -0.18 (0.19) | 0.18 (0.30) | 0.486 | 0.035 |
|  | Delayed | nTBI>HC (sad) | **Rt FP**  *(32 56 12)* | -0.18 (0.19) | 0.18 (0.30) | 0.502 | 0.028 |

Abbreviation: HC = healthy group; nTBI = non-severe traumatic brain injury group; Rt = right; Lt = left; FP = frontal pole; ACC = anterior cingulate cortex; SMG = supramarginal gyrus; ant, anterior division; RAVLT = Rey Auditory Verbal Learning Test; PIQ = Performance IQ; WCST = Wisconsin Card Sorting Test; WASI = Wechsler Abbreviated Scale of Intelligence; RCFT = Rey Complex Figure Test and Recognition Trial; ^a^p < 0.05.
