# Supplementary material for: Investigation of brain connectivity alteration in the non-severe traumatic brain injury: an emotional condition study
Source: PeerJ. 2026 Aug 5;14:e21593. doi: 10.7717/peerj.21593 (PMC13452427; doi:10.7717/peerj.21593)
Supplement: Supplementary file 5 [file peerj-14-21593-s021.docx]

*Table S5 Endogenous connectivity strength (A-matrix) from the DCM analysis for HC and nTBI groups*

| **From region** | **To region** | **Connectivity Strength** | |
| --- | --- | --- | --- |
|  |  | Healthy Group | nTBI Group |
| *mVN | mVN | -0.732 | -0.966 |
| mVN | dACC | -0.001 | - |
| mVN | RtAmyg | -0.128 | 0.134 |
| mVN | LtAmyg | -0.103 | 0.022 |
| dACC | mVN | 0.389 | -0.012 |
| *dACC | dACC | -0.980 | -1.003 |
| dACC | RtAmyg | 0.305 | -0.147 |
| dACC | LtAmyg | 0.126 | -0.028 |
| RtAmyg | mVN | -0.098 | -0.443 |
| RtAmyg | dACC | -0.075 | - |
| *RtAmyg | RtAmyg | -0.270 | -1.450 |
| RtAmyg | LtAmyg | -0.140 | 0.147 |
| LtAmyg | mVN | -0.499 | 0.170 |
| LtAmyg | dACC | 0.097 | 0.154 |
| LtAmyg | RtAmyg | -0.178 | -0.196 |
| *LtAmyg | LtAmyg | -0.925 | -0.855 |

Abbeviation: mVN = medial visual network; dACC = dorsal part of the cingulate cortex; Rt Amyg = right amygdala; Lt Amyg = left amygdala. Empty cells indicate connectivity strength with a probability of being nonzero lower than 0.95, indicating that these parameters are not statistically meaningful. * indicate intrinsic connectivity (self-connection).
