# Supplementary material for: Investigation of brain connectivity alteration in the non-severe traumatic brain injury: an emotional condition study
Source: PeerJ. 2026 Aug 5;14:e21593. doi: 10.7717/peerj.21593 (PMC13452427; doi:10.7717/peerj.21593)
Supplement: Supplementary file 6 [file peerj-14-21593-s022.docx]

*Table S6 The average connectivity strength of self-connection by the modulatory influence of positive and negative valence across experimental conditions (Diagonal of B-matrix) in both group*

|  | **Healthy Group** | | **TBI Patient Group** | |
| --- | --- | --- | --- | --- |
| **Region** | **Modulation of Positive valence** | **Modulation of Negative Valence** | **Modulation of Positive valence** | **Modulation of Negative Valence** |
| mVN | -1.290 | -0.116 | 0.589 | -0.350 |
| dACC | -0.570 | -1.204 | -1.710 | -2.435 |
| Rt Amyg | -0.603 | -1.302 | 0.594 | 1.938 |
| Lt Amyg | -0.531 | -0.459 | 0.634 | 0.205 |

The average connectivity strength of self-connection by the modulatory influence of positive and negative valence across experimental conditions (Diagonal of B-matrix) in both groups. mVN = medial Visual Network; dACC = dorsal part of the Cingulate Cortex; Rt Amyg = right amygdala; Lt Amyg = left amygdala. *Empty cells indicate a parameter with a probability of being nonzero lower than 0.95 where the parameters are not considered meaningful.
