# Supplementary material for: Investigation of brain connectivity alteration in the non-severe traumatic brain injury: an emotional condition study
Source: PeerJ. 2026 Aug 5;14:e21593. doi: 10.7717/peerj.21593 (PMC13452427; doi:10.7717/peerj.21593)
Supplement: Supplementary file 7 [file peerj-14-21593-s023.docx]

*Table S7 Between-group differences (HC vs. nTBI) in effective connectivity or endogenous connectivity strength from PEB analysis.*

| **From region** | **To region** | **Value** |
| --- | --- | --- |
| mVN | dACC | - |
| mVN | RtAmyg | 0.060 |
| mVN | LtAmyg | - |
| dACC | mVN | -0.142 |
| dACC | RtAmyg | -0.118 |
| dACC | LtAmyg | - |
| RtAmyg | mVN | -0.175 |
| RtAmyg | dACC | -0.047 |
| RtAmyg | LtAmyg | - |
| LtAmyg | mVN | 0.275 |
| LtAmyg | dACC | - |
| LtAmyg | RtAmyg | 0.072 |

Only parameters with a posterior probability > 0.95 (95%) of being different from zero are displayed. A positive value indicates greater connectivity in the nTBI group compared to HC and a negative value indicates greater connectivity in the HC group.

Abbreviation: mVN = medial Visual Network; dACC = dorsal part of the Cingulate Cortex; Rt Amyg = right amygdala; Lt Amyg = left amygdala
