# Supplementary material for: Investigation of brain connectivity alteration in the non-severe traumatic brain injury: an emotional condition study
Source: PeerJ. 2026 Aug 5;14:e21593. doi: 10.7717/peerj.21593 (PMC13452427; doi:10.7717/peerj.21593)
Supplement: Supplementary file 8 [file peerj-14-21593-s024.docx]

*Table S8 Between-group differences (HC vs. nTBI) in modulatory effects of positive and negative valance on self-connections (B-matrix) from PEB analysis.*

|  | **Region** | **Modulation of Positive valence** | **Modulation of Negative Valence** |
| --- | --- | --- | --- |
| Difference between healthy group vs TBI patient group | mVN | - | - |
|  | dACC | - | - |
|  | Rt Amyg | 0.26 | 0.42 |
|  | Lt Amyg | 0.39 | - |

Only parameters with a posterior probability > 0.95 (95%) of being different from zero are displayed. A positive value indicates greater connectivity in the nTBI group compared to HC, and a negative value indicates greater connectivity in the HC group.

Abbreviation: mVN = medial Visual Network; dACC = dorsal part of the Cingulate Cortex; Rt Amyg = right amygdala; Lt Amyg = left amygdala
