# Supplementary material for: Investigation of brain connectivity alteration in the non-severe traumatic brain injury: an emotional condition study
Source: PeerJ. 2026 Aug 5;14:e21593. doi: 10.7717/peerj.21593 (PMC13452427; doi:10.7717/peerj.21593)
Supplement: Supplementary file 9 [file peerj-14-21593-s025.docx]

Table S9 List of participants enrolled in fMRI experiment and their injury information

| **nTBI Code** | **Injury location information** |
| --- | --- |
| P101 | Bilateral frontal |
| P102 | Left frontal |
| P103 | Right frontal and temporal |
| P105 | Left frontal and bilateral temporal |
| P106 | Left parietal and occipital |
| P107 | Bilateral frontal and occipital, left temporal |
| P130 | Left frontal and right temporal |
| P132 | Bilateral frontal and parietal |
| P134 | Right parietal, frontal and temporal |
| P135 | Right frontal and left temporal |
| P136 | Left fronto-parieto-temporo-occipital |
| P138 | Right frontal |
| P142 | Bilateral frontal, right temporal and left occipital |
| P143 | Bilateral frontal, right temporal and left occipital |
| P144 | Left frontal |
| P145 | Right medial temporal and occipital |
| P146 | Bilateral frontal and right occipital |
| P147 | Left frontal and temporal |
| P149 | Right parietal and bilateral temporal |
| P150 | Bilateral frontal, parietal and temporal |

Injury types include a combination of contusions, hemorrhages, edema, parenchymal injuries, and fractures.
