# Supplementary material for: Investigation of brain connectivity alteration in the non-severe traumatic brain injury: an emotional condition study
Source: PeerJ. 2026 Aug 5;14:e21593. doi: 10.7717/peerj.21593 (PMC13452427; doi:10.7717/peerj.21593)
Supplement: Supplementary file 10 [file peerj-14-21593-s027.docx]

**STROBE Checklist – PeerJ Submission**

Manuscript Title: Investigation of Brain Connectivity Alteration in the Non-Severe Traumatic Brain Injury: An Emotional Condition Study

Author: Hazim Omar et al.

Journal: PeerJ

Study Type: Cross-sectional observational fMRI study

| Item No. | Recommendation | Page No. | Relevant text from manuscript |
| --- | --- | --- | --- |
| 1(a) | Indicate the study’s design with a commonly used term in the title or the abstract | 1 | Title and abstract specify 'Investigation of Brain Connectivity Alteration in the Non-Severe Traumatic Brain Injury: An Emotional Condition Study', identifying the observational and cross-sectional design. |
| 1(b) | Provide in the abstract an informative and balanced summary of what was done and what was found | 1-2 | Abstract provides clear background, methods, results, and conclusions highlighting connectivity alterations and correlations with cognitive outcomes. |
| 2 | Explain the scientific background and rationale for the investigation being reported | 3-5 | Introduction details the emotional and cognitive effects of nTBI and the rationale for using fMRI, CONN, and DCM to explore functional and effective connectivity. |
| 3 | State specific objectives, including any prespecified hypotheses | 5 | Objectives and hypotheses are clearly stated: to examine brain activation, FC, and EC differences in nTBI vs. healthy controls and their correlation with cognitive measures. |
| 4 | Present key elements of study design early in the paper | 6 | Methods section specifies a cross-sectional design using fMRI, FC, and DCM analyses among nTBI and control groups. |
| 5 | Describe the setting, locations, and relevant dates | 6 | Participants were recruited from Hospital Pakar Universiti Sains Malaysia; data collection and MRI sessions conducted at USM Health Campus, Kota Bharu. |
| 6(a) | Give eligibility criteria, and sources and methods of selection of participants | 6-7 | Inclusion: male participants with non-severe TBI (GCS 8–15) and healthy controls. Exclusion: prior TBI, neurological or psychiatric disorders, substance abuse, MRI contraindications. |
| 7 | Clearly define all outcomes, exposures, predictors, potential confounders, and effect modifiers | 6-8 | Outcomes include brain activation, FC, EC, and cognitive test scores; exposure is nTBI; confounders such as age and education were statistically compared. |
| 8 | For each variable of interest, give sources of data and details of methods of assessment | 8-10 | MRI and fMRI data acquisition, preprocessing, neuropsychological test administration, and parameter thresholds detailed using Philips 3T Achieva scanner and standard protocols. |
| 9 | Describe any efforts to address potential sources of bias | 6-9 | Matched control group, identical imaging protocols, exclusion of comorbidities, and statistical control for education level reduce bias. |
| 10 | Explain how the study size was arrived at | 6 | Sample of 20 nTBI and 20 HC selected based on prior similar neuroimaging studies and feasibility within TRGS-funded project. |
| 11 | Explain how quantitative variables were handled in the analyses | 10-11 | Variables analyzed using t-tests, GLM, FWE corrections, and FC/EC modeling with parametric empirical Bayes; continuous variables retained as-is. |
| 12 | Describe all statistical methods, including those used to control for confounding | 10-13 | SPSS 29 and SPM12 used for statistical analyses; FDR/FWE corrections applied; group-level contrasts and effect size (Hedges’ g) reported. |
| 13(a) | Report numbers of individuals at each stage of study | 6-7 | 40 participants enrolled (20 nTBI, 20 HC); subset completed neuropsychological tests (11 HC, 19 nTBI). |
| 14(a) | Give characteristics of study participants | 13-14 | Table 3 summarizes demographics and neuropsychological outcomes; all participants were right-handed Malay males. |
| 15 | Report numbers of outcome events or summary measures | 14-16 | Activation, FC, EC differences reported with coordinates, statistical thresholds, and correlations with cognitive scores. |
| 16 | Give unadjusted and adjusted estimates and their precision | 14-17 | Group contrasts reported as HC>nTBI and nTBI>HC; significance at pFDR<0.05; effect sizes (Hedges’ g) provided. |
| 17 | Report other analyses done—subgroups, interactions, sensitivity analyses | 18 | Condition-specific FC (sad vs fear) and correlation analyses performed; valence-specific DCM analyses compared between groups. |
| 18 | Summarise key results with reference to study objectives | 18-20 | Findings show nTBI-related reduction in visual activation and FC/EC dysregulation linked to memory and executive deficits. |
| 19 | Discuss limitations of the study | 27-28 | Limitations include small sample size, male-only cohort, cross-sectional design, and COVID-19 constraints on recruitment. |
| 20 | Give a cautious overall interpretation of results considering objectives and limitations | 26-27 | Interpretation emphasizes disrupted top-down regulation and enhanced bottom-up emotional processing in nTBI; caution noted regarding generalizability. |
| 21 | Discuss the generalisability (external validity) of the study results | 28 | Generalizability limited by homogenous male sample; recommends inclusion of female participants and larger samples in future studies. |
| 22 | Give the source of funding and the role of the funders | 29 | Funded by TRGS (MOHE Malaysia) and RUI (USM); funders had no role in design, analysis, or interpretation. |
