# Supplementary material for: Investigation of brain connectivity alteration in the non-severe traumatic brain injury: an emotional condition study
Source: PeerJ. 2026 Aug 5;14:e21593. doi: 10.7717/peerj.21593 (PMC13452427; doi:10.7717/peerj.21593)
Supplement: Supplementary file 11 [file peerj-14-21593-s028.docx]

Additional supplementary file code for categorical data and numerical data for the fMRI HC vs nTBI.sav

| Code in SPSS file | Contrasts and conditions for the activated region |
| --- | --- |
| Sad_Rt_FP | HC>PT1 Rt FP Sad |
| Fear_Rt_FP | HC>PT1 Rt FP Fear |
| Calm_Rt_FP | HC>PT1 Rt FP Calm |
| Happy_Rt_FP | HC>PT1 Rt FP Happy |
| Sad_Rt_AC | HC>PT1 Rt AC Sad |
| Fear_Rt_AC | HC>PT1 Rt AC Fear |
| Calm_Rt_AC | HC>PT1 Rt AC Calm |
| Happy_Rt_AC | HC>PT1 Rt AC Happy |
| Sad_Lt_AC | HC>PT1 Lt AC Sad |
| Fear_Lt_AC | HC>PT1 Lt AC Fear |
| Calm_Lt_AC | HC>PT1 Lt AC Calm |
| Happy_Lt_AC | HC>PT1 Lt AC Happy |
| Sad_Lt_AC_mVN | PT1>HC Lt AC Sad |
| Sad_Rt_SMG_mVN | PT1>HC Rt SMG Sad |
| Sad_Lt_SMG_mVN | PT1>HC Lt SMG Sad |
| Sad_Rt_FP_mVN | PT1>HC Rt FP Sad |
| Fear_Lt_SMG_mVN | PT1>HC Lt SMG Fear |

All the FC values for each contrast comparison above were extracted from the activated cluster into CONN toolbox as second-level covariates. These covariates which contains FC value from each participant were later exported into SPSS data for further analysis (e.g. correlation)

| Code is SPSS file | Contrasts and conditions for the activated region |
| --- | --- |
| Days_Since_Injury | Days since injury |
| GCS | Glasgow Coma Scale |
| Category | ‘1’ set for control group, ‘2’ set for nTBI group |
| Age | Age of participant |
| Edu | Formal education years |
| TBI | Type of TBI, ‘0’ for non TBI, ‘1’ for mild TBI, ‘2’ for moderate TBI. (Both ‘1’ and ‘2’ are nTBI) |
| PIQ | Performance Intelligent quotient |
| Block_design | Block design score |
| Matrix_reasoning | Matrix reasoning score |
| RAVLT_i | Rey Auditory Verbal Learning Test (immediate recall) score |
| RAVLT_d | Rey Auditory Verbal Learning Test (delayed recall) score |
| RCFT_i | Rey Complex Figure Test (immediate recall) score |
| RCFT_d | Rey Complex Figure Test (delayed recall) score |
| WCST | Wisconsin Card Sorting Test score |
| CTMT | Comprehensive Trail-Making Test score |
